# Physical Activity During Lockdowns Associated with the COVID-19 Pandemic: A Systematic Review and Multilevel Meta-analysis of 173 Studies with 320,636 Participants

**DOI:** 10.1186/s40798-022-00515-x

**Published:** 2022-10-11

**Authors:** Jan Wilke, Anna Lina Rahlf, Eszter Füzéki, David A. Groneberg, Luiz Hespanhol, Patrick Mai, Gabriela Martins de Oliveira, Johanna Robbin, Benedict Tan, Steffen Willwacher, Karsten Hollander, Julian David Pillay

**Affiliations:** 1 Institute of Occupational, Social and Environmental Medicine, Goethe University Frankfurt, Frankfurt/Main, Germany; 2 Department of Sports Science, Institute of Health, Nutrition and Sports Science, Europa-Universität Flensburg, Flensburg, Germany; 3 Masters and Doctoral Programs in Physical Therapy, Universidade Cidade de São Paulo (UNICID), São Paulo, Brazil; 4 Amsterdam Collaboration on Health and Safety in Sports, Department of Public and Occupational Health, Amsterdam Movement Sciences, Amsterdam University Medical Centers – Vrije Universiteit Amsterdam, Amsterdam, The Netherlands; 5 Institute of Biomechanics and Orthopaedics, German Sport University Cologne, Cologne, Germany; 6 Department of Mechanical and Process Engineering, Offenburg University of Applied Sciences, Offenburg, Germany; 7 Department of Sport and Exercise Medicine, Changi General Hospital, Singapore, Singapore; 8 Institute of Interdisciplinary Exercise Science and Sports Medicine, MSH Medical School Hamburg, Am Kaiserkai 1, 20457 Hamburg, Germany; 9 Department of Basic Medical Sciences, Durban University of Technology, Durban, South Africa

**Keywords:** Coronavirus, Confinements, Inactivity, Sedentary behavior, Public life restrictions

## Abstract

**Background:**

Many countries have restricted public life in order to contain the spread of the novel coronavirus (SARS-CoV2). As a side effect of related measures, physical activity (PA) levels may have decreased.

**Objective:**

We aimed (1) to quantify changes in PA and (2) to identify variables potentially predicting PA reductions.

**Methods:**

A systematic review with random-effects multilevel meta-analysis was performed, pooling the standardized mean differences in PA measures before and during public life restrictions.

**Results:**

A total of 173 trials with moderate methodological quality (modified Downs and Black checklist) were identified. Compared to pre-pandemic, total PA (SMD − 0.65, 95% CI − 1.10 to − 0.21) and walking (SMD − 0.52, 95% CI − 0.29 to − 0.76) decreased while sedentary behavior increased (SMD 0.91, 95% CI: 0.17 to 1.65). Reductions in PA affected all intensities (light: SMD − 0.35, 95% CI − 0.09 to − 0.61, *p* = .013; moderate: SMD − 0.33, 95% CI − 0.02 to − 0.6; vigorous: SMD − 0.33, − 0.08 to − 0.58, 95% CI − 0.08 to − 0.58) to a similar degree. Moderator analyses revealed no influence of variables such as sex, age, body mass index, or health status. However, the only continent without a PA reduction was Australia and cross-sectional trials yielded higher effect sizes (*p* < .05).

**Conclusion:**

Public life restrictions associated with the COVID-19 pandemic resulted in moderate reductions in PA levels and large increases in sedentary behavior. Health professionals and policy makers should therefore join forces to develop strategies counteracting the adverse effects of inactivity.

**Supplementary Information:**

The online version contains supplementary material available at 10.1186/s40798-022-00515-x.

## Key Points

- Meta-analytic pooling of 173 studies revealed that physical activity decreased and sedentary behavior increased during lockdowns associated with the COVID-19 pandemic.
- According to moderator analyses, both observations were largely independent from variables such as age, sex, body mass index, or health status.

## Introduction

As of July 28, 2022, the COVID-19 pandemic has caused an estimated 6.4 million deaths in more than 200 affected countries [1]. To stop the contagion, many governments imposed public life restrictions such as curfews, business closures, or bans on social gatherings and mass events [2, 3]. While these measures mitigated the viral spread [2, 3], they limited spaces and opportunities to engage in physical activity (PA) through, for example, closed gyms, parks, and sports clubs [4]. At the same time, lockdown measures might have increased sedentary behavior (SB), e.g., due to higher sitting and screen times. Both, PA and SB are of particular concern as a large body of evidence underscores the value of PA as a cornerstone of health and the detrimental effects of SB. For instance, being active on a regular basis has been demonstrated to reduce the risk of coronary heart disease, stroke, metabolic syndrome, type 2 diabetes, breast and colon cancer, and depression [5, 6]. On the contrary, physical inactivity leads to 9% of premature mortality [5], raising national healthcare expenditures by up to 4.6% [7]. Also, SB has been established as a major health risk [8]. In addition to its general health effects, PA is protective against COVID-19. A recent analysis of 65,361 adults demonstrated lower risk for hospitalization (RR 0.66, 95% CI 0.63 to 0.70), intensive care unit (ICU) admission (RR 0.59, 95% CI 0.52 to 0.66), required ventilation (RR 0.55, 95% CI 0.47 to 0.64), and death (RR 0.58, 95% CI 0.50 to 0.68) in individuals with high vs. low PA levels [9].

Considering the pivotal role of PA at the individual and public health levels, a plethora of research has been undertaken to gauge the consequences of public life restrictions on movement behavior. In a systematic review of 66 studies, Stockwell et al. [10] concluded that PA levels decreased during lockdown periods, in line with findings of other papers [11, 12]. However, to date, no quantitative data synthesis is available and the factors driving decreases in PA are unknown. Therefore, the objectives of this study were: (1) to summate the effects of governmental-enforced public life restrictions on PA markers; and (2) to identify potential moderators of this association.

## Methods

A systematic review with multilevel meta-analysis and a moderator analysis were performed. The article adheres to the PERSiST (implementing PRISMA in Exercise, Rehabilitation, Sport medicine and SporTs science) guidance statement [13] and follows the recommendations for ethical publishing of systematic reviews [14]. The study was registered in the PROSPERO database (CRD42021238793).

### Literature Search

In September 2021, two independent investigators (KH, SW) performed a systematic literature search. Articles pertaining to the research question were identified using PubMed, Web of Science and Google Scholar. The search strategy used in PubMed was: ‘(“physical activity”) AND (SARS-CoV2 OR COVID-19 OR coronavirus) AND (restrictions OR confinement OR lockdown OR pandemic).’ To complement database searches, the reference lists of all included studies were screened manually in order to identify additional potentially eligible papers [15]. Cross-sectional, cohort, case–control, and other observational studies were eligible for inclusion if reporting continuous dependent data on PA pre- (PA_baseline_) and during (PA_restrictions_) public life restrictions relating to the COVID-19 pandemic. Articles reporting non-original data (e.g., reviews, study protocols, commentaries), qualitative studies and intervention trials were excluded. We considered studies with male and female participants of all ages, with and without medical conditions. Articles had to be written in English and published in a peer-reviewed journal. Disagreements between the two investigators regarding study eligibility were resolved by means of a third investigator.

### Data Extraction

Four independent investigators [GMO, EF, PM, ALR] extracted the following data: study design, sample size, participant characteristics, measured outcomes, and results (mean/median of PA_baseline_ and PA_restrictions_, standard deviation [SD]/interquartile range [IQR], mean/median changes in PA and SD/IQR; same for SB markers such as sitting time or screen time).

### Data Synthesis and Statistics

For each study, the changes between PA_baseline_ and PA_restrictions_, and between SB_baseline_ and SB_restrictions_ as well as their correlation (if the data set was available) were calculated. If the correlation coefficient was not reported or the dataset was not available, it was imputed using the formula ${\text{Corr}} = {\text{SD}}_{{{\text{baseline}}}}^{2} + {\text{ SD}}_{{{\text{restrictions}}}}^{2} {-}{\text{ SD}}_{{{\text{change}}}}^{2} /{2} \times {\text{SD}}_{{{\text{baseline}}}} \times {\text{SD}}_{{{\text{restrictions}}}}$ [16]. Where no imputation was possible (e.g., due to missing SD_change_), a conservative Corr value of 0.5 was assumed [16], which fits with the known correlations of the included studies. Following the recommendations of the Cochrane handbook [15], missing SD_change_ data were imputed as ${\text{SD}}_{{{\text{change}}}} = \surd \left( {{\text{SD}}_{{{\text{baseline}}}}^{2} + {\text{SD}}_{{{\text{restrictions}}}}^{2} } \right){-}\left( {{2} \times {\text{Corr}} \times {\text{SD}}_{{{\text{baseline}}}} \times {\text{SD}}_{{{\text{restrictions}}}} } \right)$, where Corr = 0.5. Medians and interquartile ranges were converted into means and standard deviations using the Box–Cox method of McGrath et al. [17].

The following moderators were coded as categorical data: study design (cross-sectional or longitudinal), age, sex (female or male), body mass index (BMI, low or high), activity level (inactive or active), origin (Africa, Asia, Australia, Europe, North America, or South America), quality of life (high or low), stringency of public life restrictions (high or low) health status (healthy or diseased), and assessment method (subjective or objective). With regard to the study design, studies asking for PA before and during the pandemic at one time point (e.g., using questions in one survey) were classified as cross-sectional and only studies assessing PA at multiple time points were defined as longitudinal. For age, sample means were used to classify the participants as children and adolescents (< 18 years), adults (18 to 64 years), or old adults (> 64 years). If reported, BMI was used to identify samples with normal (< 25 kg/m^2^) or overweight (> 25 kg/m^2^) participants. To code the activity level, we used the World Health Organization (WHO) recommendations on PA [18] to classify samples as inactive (not complying with guidelines) or active (complying with guidelines). With regard to quality of life, we used the composite score proposed by Peiró-Palomino and Picazo-Tadeo [19] which is based on the Better Life Index of The Organisation for Economic Co-operation and Development (OECD). The index includes 10 indicators (housing, income, jobs, community, education, environment, civic engagement, health, safety, and work-life balance), and it is available for the 35 OECD countries and South Africa, Russia, and Brazil. The resulting score ranges between 0 and 1 with larger values representing higher quality of life. The composite score based on OECD Better Life Index was individually calculated for each study and the origin of the respective sample. Values below 0.5 were classified as low. Finally, to quantify the stringency of governmental containment measures, the COVID-19 stringency index [20] was used. The composite measure is generated using nine governmental response indicators (school/workplace/public transport closures, public event cancellation, public gathering bans, stay-at-home orders, public information campaigns, internal movement restrictions, and international travel restrictions). The resulting score spans 0 to 100 with larger values representing higher stringency. The COVID-19 stringency index was calculated for the specific lockdown period of each study, if reported. Values below 50 were classified as low.

The choice of the tested moderators was based on three criteria [21]. First, they had to be clearly reported in at least five studies. Second, variation between the levels of a moderator was required. For instance, if all studies would have stated the sex of the participants, a moderator analysis would have been impossible if only men were included in these studies. Third, there had to be a plausible reasoning as to how a moderator would influence changes in PA. For instance, it may be assumed that older adults changed their movement behavior to a greater extent than younger adults due to increased fears of acquiring COVID-19 infection.

A multilevel meta-analysis with a robust random effects meta-regression model [22] was used to pool the standardized mean differences (SMD) and 95% confidence intervals (CI) between PA_baseline_ and PA_restrictions_, and between SB_baseline_ and SB_restrictions_. The dependency in effect size (ES) estimates in case of multiple outcome measures in the same study (e.g., min/weeks and steps/week) was taken into account by nesting the term ‘study’ as a random factor in the model. Potential moderators were identified with a twofold approach: (1) estimating the significance of each level, also by means of evaluating the inclusion or not of the null value within the 95% CI [23]; and (2) testing for differences between the respective levels [24, 25]. The between-study variance component was determined by means of Tau^2^, using the method-of-moments estimate. The within-study variance (more than one dependent effect size) was determined by omega^2^ (ω^2^) [22]. Resulting pooled ES was interpreted as small (SMD = 0.2 to 0.49), moderate (SMD = 0.50 to 0.79), or large (SMD =  ≥ 0.8) [26]. *p* values < 0.05 were considered significant. The software employed was R (R Foundation for Statistical Computing, Vienna, Austria) using packages meta [27] and robumeta (version 2.021, [28]).

### Risk of Bias Assessment

The risk of bias of the studies was assessed using an adapted Downs and Black checklist (Additional file 1: Table S1), which has been shown to exhibit high reliability and validity in the assessment of non-randomized studies [29, 30]. The maximum score was 16. Two independent investigators [JDP, GMO] performed the quality scoring, resolving disagreement using a third rater. In addition, to identify reporting bias, we visually inspected funnel plots (SMD against standard errors) with optional sensitivity analyses excluding outliers if at least 10 ES were available [31].

## Results

**Fig. 1:**
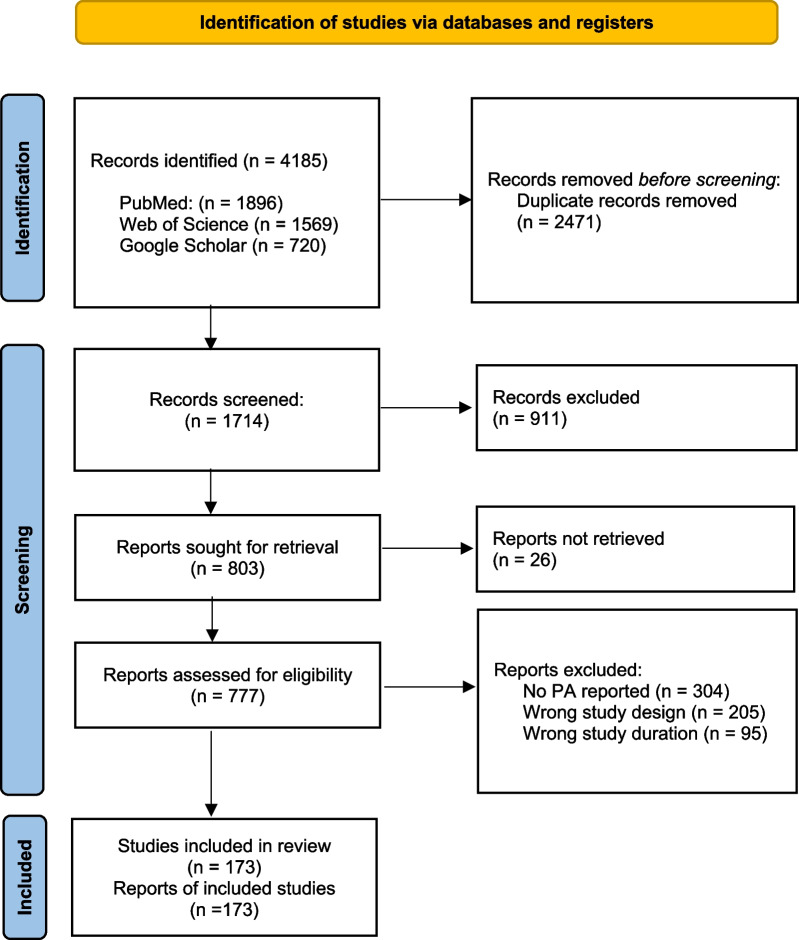
Flowchart of the literature search

The flow of the literature search is displayed in Fig. 1. Database queries returned a total of 4,185 articles, 173 of which were considered eligible for this study (Additional file 1: Tables S2, S3).

### Study Characteristics

The 173 papers collectively included 320,636 participants. Most studies (65.9%, *n* = 114) included adults, while children and adolescents (15.6%, *n* = 27) or older adults (14.5% *n* = 25) were less frequently targeted. Twenty-nine (16.8%) studies focused on individuals with chronic diseases. More than the half of the investigations were performed in Europe (51.4%, *n* = 89), followed by Asia (16.2% *n* = 28), North America (12.7%, *n* = 22), South America (7.5%, *n* = 13), Africa, and Australia (both 2.9%, *n* = 5). Regarding the study design, slightly more cross-sectional studies (54.0%, *n* = 93) than longitudinal studies (46.0%, *n* = 80) were identified. Subjective PA measures were more frequent (*n* = 143) than objective measures (*n* = 33).

### Risk of Bias

**Fig. 2:**
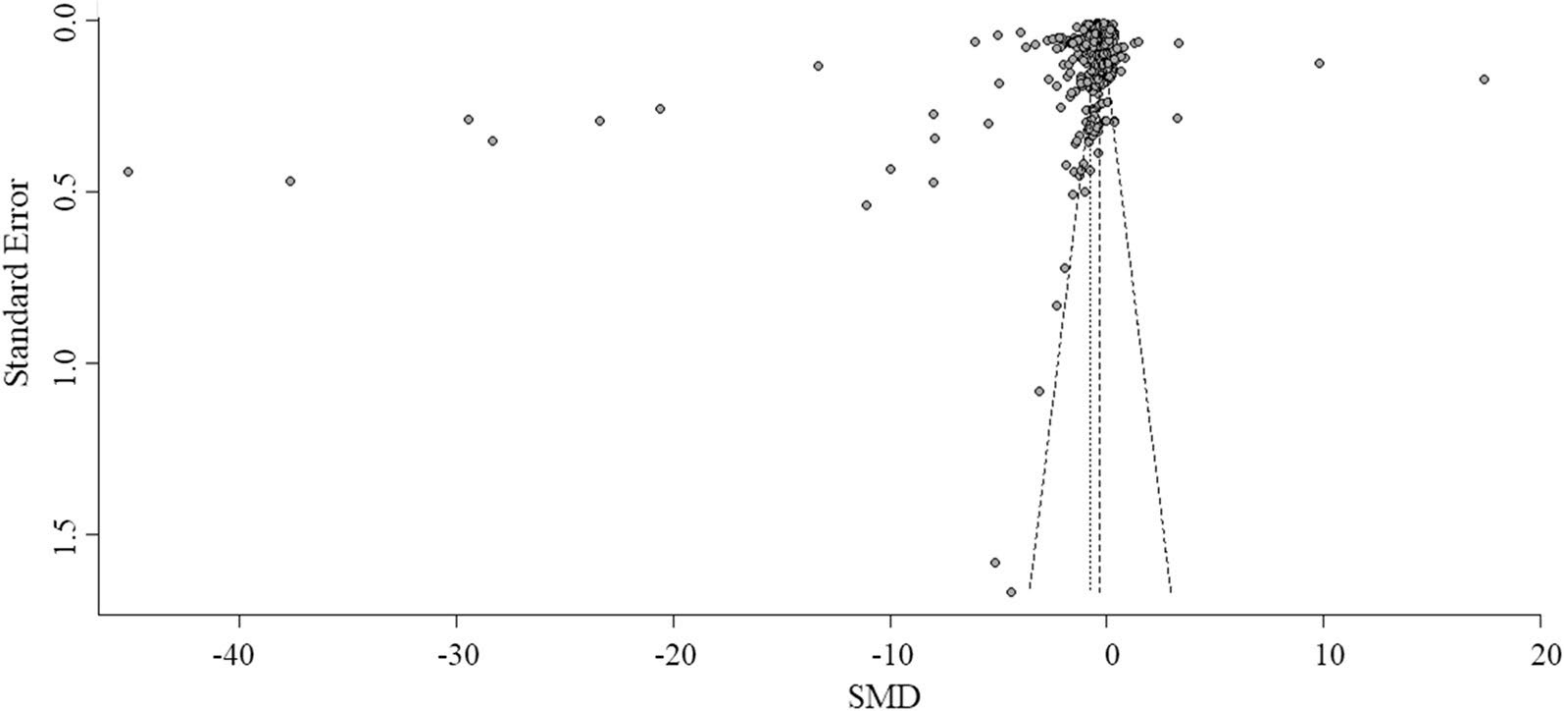
Funnel plot of changes in total physical activity. Note the slight lack of smaller studies with positive standardized mean difference (SMD) on the right side of the plot

Investigators agreed in 2712 (97.4%) of the 2784 criteria scored by means of the Downs and Black checklist. All initial disagreements could be resolved. Ratings of the included studies ranged from 4 to 16 out of 16 (Additional file 1: Table S4) and on average, the risk of bias was classified as low (12.9 ± 2.1 points). Most studies had precise reporting (sub-score: 7.0 ± 1.3 out of 8 points) and moderate to high internal validity (bias: 3.6 ± 0.7 out of 4 points, confounding: 1.3 ± 0.7 out of 2 points). However, only about one quarter of the studies had representative samples and hence high external validity. Inspection of funnel plots (Fig. 2) revealed a slight asymmetry with a lack of small studies reporting PA decreases. Yet, in view of the high number of papers located around the summary effect estimate, its influence may be limited.

### Meta-analysis

We found a moderate reduction in total PA (SMD − 0.65, 95% CI − 1.10 to − 0.21, *p* = .004, 172 studies, 605 ES, Table 1). Although smaller in magnitude, PA decreases also occurred when considering only walking (SMD − 0.52, (− 0.29 to − 0.76), *p* < .001, 39 studies, 63 ES) as well as activities of light (SMD − 0.35, 95% CI: − 0.09 to − 0.61, *p* = .013, 13 studies, 15 ES), moderate (SMD − 0.33, 95% CI − 0.02 to − 0.6, *p* = .04, 45 studies, 71 ES,), and vigorous (SMD − 0.33, 95% CI − 0.08 to − 0.58, *p* = .01, 43 studies, 72 ES) intensity. Contrary to PA, there was a large increase in SB (SMD 0.91, 95% CI: 0.17 to 1.65, *p* = .02, 71 studies, 114 ES). Sensitivity analyses excluding imputed data (needed in 141 out of 173 studies) and studies with high risk of bias yielded similar results, confirming the robustness of the main analysis.

**Table 1:** Results of the meta-analysis

Outcome	Studies (ES)	SMD (95%CI)	*p* value	Tau^2^/Omega^2^
Total PA	172 (605)	− 0.65 (− 1.10 to − 0.21)	.004	.34/0
Walking	39 (63)	− 0.52 (− 0.29 to − 0.76)	< .001	.13/0
Light PA	13 (15)	− 0.35 (− 0.09 to − 0.61)	.013	.15/0
Moderate PA	45 (71)	− 0.33 (− 0.02 to − 0.63)	.04	.15/0
Vigorous PA	43 (72)	− 0.33 (− 0.08 to − 0.58)	.01	.09/0
Sedentary behavior	70 (113)	0.91 (0.17 to 1.65)	.02	.37/0

*PA* Physical activity, *ES* Effect sizes, *SMD* Standardized mean difference, *CI* Confidence interval

### Moderator Analysis

Meta-regression (Table 2) showed no influence of sex, BMI, health status, and assessment method on PA. Although higher ES was found for adults (vs. children and adolescents, or old adults), active (vs. inactive) individuals, countries with low (vs. high) quality of life, and countries with high (vs. low) lockdown stringency, the respective between-level comparisons were not significant (i.e., the null value was found inside the 95% CI). Two further moderators were identified. While PA decreases affected all continents, no reduction was found for Australia when compared to the reference (Europe). In addition, studies with cross-sectional design were associated with higher ES compared to longitudinal studies.

**Table 2:** Results of the moderator analysis

Moderator	Studies (ES)	Mean estimate (95% CI)	Tau^2^/Omega^2^	Difference between levels
*Sex*				
Male	34 (78)	− 1.78 (− 4.04 to 0.47)	1.10/0	*t* = 1.22, *p* = .23
Female	38 (87)	− 1.34 (− 2.90 to 0.23)		
*Age*				
Children and adolescents (< 18 yrs.)	27 (98)	− 0.40 (− 0.78 to − 0.02)	0.4/0	Intercept
Adults (18–64 yrs.)	114 (392)	− 0.83 (− 1.51 to − 0.15)		*t* = − 1.10, *p* = .28
Old adults (≥ 65 yrs.)	25 (74)	− 0.36 (− 0.51 to − 0.21)		*t* = 0.17, *p* = .87
*Body mass index*				
Normal (< 25 kg/m^2^)	42 (150)	− 1.02 (− 1.92 to − 0.11)	0.72/0	*t* = − 0.73, *p* = .47
High (≥ 25 kg/m^2^)	31 (122)	− 1.29 (− 2.68 to 0.11)		
*Pre-activity level*				
Inactive	15 (58)	− 0.38 (− 0.62 to − 0.15)	0.50/0	*t* = − 1.1, *p* = .27
Active	71 (295)	− 0.92 (− 1.86 to 0.02)		
*Health status*				
Healthy/mixed	147 (511)	− 0.68 (− 1.20 to − 0.16)	0.35/0	*t* = 0.54, *p* = .59
Diseased	29 (96)	− 0.51 (− 0.88 to − 0.14)		
*Quality of life*				
Low (WBI < 0.5)	50 (155)	− 1.33 (− 3.03 to 0.38)	0.46/0	*t* = − 1.09, *p* = .28
High (WBI ≥)	80 (298)	− 0.38 (− 0.59 to − 0.16)		
*Lockdown stringency*				
Low (SI < 50)	16 (77)	− 0.41 (− 0.75 to − 0.32)	0.38/0	*t* = 0.63, *p* = .53
High (SI ≥ 50)	143 (497)	− 0.83 (− 1.73 to 0.08)		
*Location*				
Africa	5 (38)	− 1.10 (− 1.46 to − 0.74)	0.32/0	*t* = − 0.55, *p* = .59
Australia	5 (26)	− 0.01 (− 0.18 to 0.17)		*t* = 1.88, *p* = .06*
Asia	28 (89)	− 0.33 (− 0.51 to − 0.14)		*t* = 1.16, *p* = .25
Europe	88 (298)	− 0.84 (− 1.71 to 0.02)		Intercept
North America	22 (84)	− 0.25 (− 0.36 to − 0.14)		*t* = 1.35, *p* = .18
South America	13 (34)	− 0.39 (− 0.49 to − 0.30)		*t* = 1.03, *p* = .31
*Study design*				
Cross-sectional	93 (341)	− 0.95 (− 1.70 to − 0.20)	0.34/0	*t* = 1.81, *p* = .07*
Longitudinal	80 (264)	− 0.25 (− 0.35 to − 0.16)		
*Assessment*				
Objective	33 (111)	− 0.50 (− 0.70 to − 0.30)	0.35/0	*t* = 0.68, *p* = .50
Subjective	143 (492)	− 0.70 (− 1.23 to − 0.16)		

*SMD* Standardized mean difference, *CI* Confidence interval, *yrs* Years, *SI* Stringency index, *WBI* Well-being index

*p < .1

## Discussion

After the worldwide imposition of lockdown measures aimed at curbing the spread of the COVID-19 pandemic, there have been several warnings that PA levels could decrease due to the restrictions on individual movement and exercise [4, 32, 33]. The present systematic review with meta-analysis provides firm evidence of substantial PA declines during restrictions. This finding is in line with the results of previous reviews that did not include quantitative data synthesis [10–12]. To our knowledge, our work is not only distinct from previous studies in for the first time providing a pooled effect estimate: while Stockwell et al. [10] found 60 eligible articles, we were able to almost triple this number with 173 trials including over 320,000 participants. Although our search period was almost one year longer (until October 2020 for Stockwell et al. [10], until September 2021 in our study), this large difference impressively underlines the strong focus of current research on PA and the COVID-19 pandemic.

The general recommendation of “move more, sit less” for health is underpinned by an increasing body of evidence [8]. Against this background, the large increase in SB that we document is of particular public health relevance. Higher levels of SB are detrimentally associated with a variety of adverse health outcomes, including all-cause and cardiovascular disease mortality, cardiovascular disease, type 2 diabetes and certain cancers [34]. Also, higher levels of PA and lower levels of SB have been shown to be negatively linked to instrumental activities of daily living [35] as well as muscle strength and power in older adults [36]. Similar patterns were observed for cognitive function in older adults [37].

The importance of PA as a pivotal contributor to health has repeatedly been demonstrated, as PA plays a significant role in the prevention and management of various chronic diseases [5, 32, 33]. Although pandemic-related public life restrictions are typically limited in duration, even short-term physical inactivity with increases in SB can have significant adverse effects. Only 5 days of bedrest have been demonstrated to cause arterial stiffening, impaired endothelial function and elevated diastolic blood pressure [38]. In another study, a two-week step reduction induced a decrease in insulin sensitivity and cardiorespiratory fitness while increasing body and liver fat as well as low density lipoprotein concentrations [39]. While such changes seem mostly reversible in younger individuals, they are less so for the elderly and individuals with metabolic conditions [40]. In addition to having a somatic impact, inactivity also affects psychological well-being. Public life restrictions led to a threefold increase in depression risk markers [41] and, in addition, a systematic review of systematic reviews found a higher prevalence of anxiety, stress, stigma, and post-traumatic stress syndrome during lockdowns [42]. Before the pandemic, it had already been shown that PA (i.e., during leisure time) correlates with positive affect, life satisfaction [43] and well-being [44]. Of note, analyzing data collected during the pandemic, Cross et al. [45] demonstrated a clear positive association between the level of PA and mental well-being in a large cohort of American adults. As a consequence, it seems of paramount importance to identify ways to maintain or even enhance PA during restrictions. One effective method to achieve this while social distancing is through tele-exercise, which has recently been shown to be efficacious in this context [46]. In addition, digital technologies (i.e., telehealth) could be used to promote and counsel on PA without in-person contact. Pinto et al. [32] showed that home-based training combined with online PA counseling can increase PA and decrease SB in patients with cardiovascular disease.

According to our sub-analyses, the decreases in PA affected movement behavior at all intensities to a similar degree. Interestingly, relative to the reductions in light, moderate, and vigorous activities, the pooling of all PA measures yielded a large ES. This may be due to the fact that most studies providing step counts did not stratify for intensity and hence were only included in the main analysis. Another relevant finding is that the moderator analysis did not identify significant associations with a variety of tested predictors. The lack of effects regarding variables such as age, sex, body mass index, or health status may mean that the restrictions made it highly difficult to evade their impact. A substantial portion of the studies on PA changes during lockdowns was based on self-reported data, and it has been suggested that the reported PA reductions could be perceived rather than actual [47]. Interestingly, our moderator analysis demonstrated that both objective and subjective outcomes generated similar findings. However, regardless of the assessment method, there was a pronounced difference between cross-sectional (higher ES) and longitudinal (smaller ES) studies. This observation reinforces the need for repeated or continuous assessments during lockdowns as these may more reliably cover changes over time.

Some limitations merit consideration. The focus of our review was on changes in PA during lockdowns. As SB is closely linked to PA and many papers reported both, we extracted SB data too. This means that explicitly searching for papers on SB during lockdowns may have yielded some few additional papers not covered by our search. However, due to the high number of studies on SB identified, we believe that the pooled change reported here is robust. In our analysis, we used the SMD as the effect estimate. This was necessary because the included studies used highly variable measures, definitions and time spans of PA. While using the SMD allowed us to achieve a very large sample of studies, the mean difference—although referring to a small subset of studies—may have yielded valuable additive information as it can provide more informative numbers (e.g., decrease in moderate PA in min/week). Finally, we used the Better Life Index as a surrogate of a country’s living quality. As not all studies included in the analysis managed to recruit a representative sample and as the index does not account for different individuals within the same country, this part of the moderator analysis may need to be interpreted with some caution.

## Conclusions

PA levels of all intensities have decreased, while SB has increased during the COVID-19 pandemic lockdowns. These findings run against the frequently recommended “move more, sit less” paradigm for health. Changes in PA were largely independent of markers such as age, sex, BMI, or health status. Considering the beneficial physical and psychological health benefits of PA, researchers and policy makers should strive to devise interventions aimed at promoting PA and reducing SB when public life is restricted.

## Supplementary Information

**Additional file 1. Table S1.** Checklist for study risk of bias assessment (adapted from Downs & Black). **Table S2.** Characteristics of the included studies. Age is reported as mean ± SD or range (youngest-oldest age) or median (single value). We listed eligible criteria if none of these metrics were reported (e.g., >, < age). **Table S3.** Reference list of included studies. **Table S4.** Ratings of study risk of bias (adapted Downs & Black checklist).

## Data Availability

Not applicable.

## Abbreviations

BMI: Body mass index
CI: Confidence interval
ES: Effect size
ICU: Intensive care unit
IQR: Interquartile rate
OECD: Organisation for Economic Co-operation and Development
PA: Physical activity
RR: Relative risk
SB: Sedentary behavior
SMD: Standard mean difference
WHO: World Health Organization
