# Supplementary material for: Physical Activity During Lockdowns Associated with the COVID-19 Pandemic: A Systematic Review and Multilevel Meta-analysis of 173 Studies with 320,636 Participants
Source: Sports Med Open. 2022 Oct 11;8:125. doi: 10.1186/s40798-022-00515-x (PMC9551244; doi:10.1186/s40798-022-00515-x)
Supplement: Supplementary file 1 — Additional file 1. Table S1. Checklist for study risk of bias assessment (adapted from Downs & Black). Table S2. Characteristics of the included studies. Age is reported as mean ± SD or range (youngest-oldest age) or median (single value). We listed eligible criteria if none of these metrics were reported (e.g., >, < age). Table S3. Reference list of included studies. Table S4. Ratings of study risk of bias (adapted Downs & Black checklist). [file 40798_2022_515_MOESM1_ESM.docx]

**Supplementary Materials**

**Suppl. Tab. 1.** Checklist for study risk of bias assessment (adapted from Downs & Black)

| **Criterion** | **Comments** |
| --- | --- |
| **Reporting** | |
| *Is the hypothesis/aim/objective of the study clearly described? (yes: 1/no: 0)* |  |
| *Are the main outcomes to be measured clearly described in the Introduction or Methods section? (yes: 1/no: 0)* | If the main outcomes are first mentioned in the results section, the question should be answered no. |
| *Are the characteristics of the patients included in the study clearly described? (yes: 1/no: 0)* | Inclusion/exclusion criteria need to be given, main characteristics (e.g., age, sex, and health status) need to be given. |
| *Are the distributions of relevant confounders clearly described? (eg. age, BMI, gender, health status, income, or well-being) (yes:1/no:0)* | In both cross-sectional and longitudinal studies relevant confounders are described. |
| *Are the main findings of the study clearly described? (yes: 1/no: 0)* | Simple outcome data (including denominators and numerators) should be reported for all major findings so that the reader can check the major analyses and conclusions. Specifically, the mean and SD (or 95% CI) of PA or median and percentile before and during the lockdown should be reported. |
| *Does the study provide estimates of the random variability in the data for the main outcomes? (yes: 1/no:0)* | In non-normally distributed data, the inter-quartile range of results should be reported. In normally distributed data the standard error, standard deviation or confidence intervals should be reported. If the distribution of the data is not described, it must be assumed that the estimates used were appropriate and the question should be answered yes. |
| *Have the characteristics of patients lost to follow-up been described? (yes:1/no:0)* | **Only for longitudinal studies:** Answered “yes” when characteristics of the patients lost to follow-up were described. |
| *Have actual probability values been reported for the main outcomes except where the probability value is less than 0.001? (yes:1/no:0)* |  |
| **External validity** | |
| *Were those subjects who were prepared to participate representative of the entire population from which they were recruited? (yes:1/no:0)* | The proportion of those asked who agreed should be stated. Further the authors should mention in the paper that the included participants were representative for the investigated population. |
| **Internal validity – bias** | |
| *If any of the results of the study were based on “data dredging”, was this made clear? (yes:1/no:0)* | Any analyses that had not been planned at the outset of the study should be clearly indicated. If no retrospective unplanned subgroup analyses were reported, then answer yes. |
| *Is the time period referred to before lockdowns identical to that during lockdowns? (yes:1/no:0)* | If the time periods of PA assessment were identical, this is scored yes. If different lengths of follow-up were adjusted for, for example, using survival analysis, the answer should be yes. Studies, where differences in follow-up are ignored should be scored “no”. |
| *Were the statistical tests used to assess the main outcomes appropriate? (yes:1/no:0)* | The statistical techniques used must be appropriate to the data (e.g. t-test / Wilcoxon test for dependent samples). For example, nonparametric methods should be used for small sample sizes. Where little statistical analysis has been undertaken but where there is no evidence of bias, the question should be answered yes. If the distribution of the data (normal or not) is not described, it must be assumed that the estimates used were appropriate and the question should be answered yes. |
| *Were the physical activity measures used accurate (valid and reliable)? (yes:1/no:0)* | For studies where the outcome measures are clearly described, the question should be answered yes. For studies which refer to other work or that demonstrates the outcome measures are accurate, the question should be answered as yes. No matter what kind of assessment the study used, an adequate reference or evaluation method must be mentioned in the paper to get a “yes”. |
| **Internal validity- confounding (selection bias)** | |
| *Participant sampling described (yes:1/no:0)* | If assignment was based on randomised selection, census, or use of a representative sample register, one point is given. If not, it should be answered no=0. |
| *Was there adequate adjustment for confounding in the analyses from which the main findings were drawn? (yes:1/no:0)* | If the effect of the main confounders was not investigated or confounding was demonstrated but no adjustment was made in the final analyses the question should be answered as no. |
| *Power* | |
| *Did the study have sufficient power to detect a clinically important effect where the probability value for a difference being due to chance is less than 5%? (yes:1/ no:0)* | If a sample size calculation is provided or significant effects in the main outcome were detected, this is answered scored with a point. In papers, where the sample size calculation was based on another main outcome than physical activity, only a “yes” can be given if the physical activity results show significant differences. |

**Suppl. Tab. 2.** Characteristics of the included studies. Age is reported as mean ± SD or range (youngest-oldest age) or median (single value). We listed eligible criteria if none of these metrics were reported (e.g., >, < age).

| **Study** | **Design** | **Country** | **Participants** | **PA Outcome** |
| --- | --- | --- | --- | --- |
| Abid et al. [1] | CS | Tunisia | n = 100 children (48 females), 8.7 ± 3.3 years | Survey |
| Acs et al. [2] | CS | Hungary | n = 827 students (648 females), 25.3 ± 8.1 years | Survey |
| Aegerter et al. [3] | L | Switzerland | n = 76 adults and office workers (54 females), 42.7 (21.8 - 62.7) years | Survey |
| Aguilar-Farias et al. [4] | CS | Chile | n = 3157 toddlers and preschoolers (1560 females), 3.1 ± 1.4 years | Survey |
| Al-Musharaf et al. [5] | L | Saudi Arabia | n = 297 young female adults (297 females), 20.7 ± 1.4 years | Survey |
| Alonso-Martinez et al. [6] | L | Spain | n = 268 preschoolers (125 females), 4.3 ± 0.8 years | Accelerometers |
| Amini et al. [7] | CS | Iran | n = 670 adults (518 females), 29.2 ± 9.5 years | Survey |
| Ammar et al. [8] | CS | N/A | n = 1047 adults (484 females), >18 years | Survey |
| Assaloni et al. [9] | CS | Italy | n = 154 type 1 diabetes patients (70 females), 44.8 ± 12.5 years | Survey. Activity Tracker |
| Bann et al. [10] | L | United Kingdom | n = 13283 (6696 females), age not reported | Survey |
| Barkley et al. [11] | CS | United States | n = 389 university students and employees (298 females), 26 ± 8.9 years | Survey |
| Barone Gibbs et al. [12] | L | United States | n = 111 shelter-at-home employees (77 females), 45.4 ± 12.3 years | Survey |
| Bartlett et al. [13] | L | Australia | n = 1671 adults (1218 females), >50 years | Survey |
| Beck et al. [14] | CS | United States | n = 145 children (80 females), age not reported | Survey |
| Belgen et al. [15] | CS | Turkey | n = 104 postmenopausal women (104 females), 59 ± 6.6 years | Survey |
| Berengüi et al. [16] | CS | Denmark | n = 1019 adults (487 females), 35.3 ± 14.2 years | Survey |
| Bertrand et al.[17] | CS | Canada | n = 125 university students (95 females), age not reported | Survey |
| Bogataj Jontez et al. [18] | L | Slovenia | n = 38 adults (14 females), 36.3 ± 10.1 years | Survey |
| Bourdas et al. [19] | CS | Greece | n = 8495 adults (5242 females), 37.2 ± 0.2 years | Survey |
| Branquinho et al. [20] | CS | Portugal | n = 5746 adults (3710 females), 48.5 ± 14.3 years | Survey |
| Browne et al. [21] | L | Brazil | n = 35 hypertensive older adults (23 females), 65.6 ± 3.8 years | Survey, accelerometer |
| Buoite Stella et al. [22] | CS | Italy | n = 400 adults (277 females), 35 ± 15 years | Survey, smart technology devices |
| Calcaterra et al. [23] | CS | Italy | n = 280 children with type 1 diabetes (116 females), 11.8 ± 3.3 years | Survey |
| Calvo et al. [24] | CS | Spain, other countries | n = 169 basketball players (47 females), 24.8 ± 6.4 years | Survey |
| Castaneda-Babarro et al. [25] | CS | Spain | n = 5546 adults (1746 females), 42.7 ± 10.4 years | Survey |
| Cheval et al. [26] | L | France, Switzerland | n = 110 adults (76 females), 43 ± 19 years | Survey |
| Chouchou et al. [27] | CS | Reunion | n = 400 adults (223 females), 29.8 ± 11.5 years | Survey |
| Coughenour et al. [28] | CS | United States | n = 194 college students (140 females), 25.1 ± 7.8 years | Survey |
| Curtis et al. [29] | L | Australia | n = 61 adults (40 females), 41.3 ± 5.8 years | Fitbit Charge 3, Survey |
| Czenczek- Lewandowska et al. [30] | CS | Poland | n = 506 young adults (355 females), 24.7 ± 4.2 years | Survey |
| Davy et al. [31] | CS | South Africa | n = 1048 adults (767 females), 27 (21-42) years | Survey |
| de la Vega et al. [32] | CS | International | n = 1079 exercise addiction, athletes (number of females not reported), 32.9 ± 11.7 years | Survey |
| DeJong et al. [33] | CS | International | n = 1147 athletes (256 females), age not reported | Survey |
| Delisle Nystrom et al. [34] | L | Sweden | n = 81 preschoolers (23 females), 4.0 ± 0.5 years | Survey |
| Di Sebastiano et al. [35] | L | Canada | n = 2338 adults (2109 females), 35-54 years | Smartphone Application |
| Di Stefano et al. [36] | CS | Italy | n = 149 neuromuscular disease patients and controls (number of females not reported), Disease patients 57.3 ± 13.7; healthy controls 56 ± 6.8 years | Survey |
| Di Stefano et al. [37] | CS | Italy | n = 261 adults with diagnosed migraine (227 females), 45 (34.5–53) years | Survey |
| Ding et al. [38] | L | China | n = 815 adults (530 females), age not reported | Smartphone Application |
| Dobrowolski et al. [39] | CS | Poland | n = 183 adults (number of females not reported), 33 ± 11 years | Survey |
| Dogas et al. [40] | CS | Croatia | n = 2498 adults (1989 females), age not reported | Survey |
| Dun et al. [41] | CS | China | n = 10340 college students (8292 females), 19 ± 1 (17-27) years | Survey |
| Dunton et al. [42] | CS | United States | n = 268 adults (215 females), 34 ± 12.2 years | Smartphone Application |
| Ellis et al. [43] | CS | International | n = 2004 adults (807 females), 30.5 (18-99) years | Survey |
| Elran-Barak et al. [44] | CS | Israel | n = 315 chronic medical conditions patients (189 females), 18-65 years | Survey |
| Esain et al. [45] | L | Spain | n = 58 older adults (45 females), 76.2 ± 6.0 years | Survey |
| Fernandez-Garcia et al. [46] | L | Spain | n = 18 older adults (11 females), 78.4 ± 5.4 years | Survey |
| Feter et al. [47] | CS | Brazil | n = 2314 adults (1772 females), >18 years | Survey |
| Flanagan et al. [48] | CS | International | n = 7753 adults (1551 females), 51.2 ± 0.17 years | Survey |
| Francisco-Pascual et al. [49] | L | Spain | n = 245 adults with an ICM (129 females), 64.5 (45.0–79.0) years | Implantable cardiac monitor |
| Franco et al. [50] | CS | Italy | n = 310 adults (223 females), 46.9 ± 11.30 years | Survey |
| Franco et al. [51] | L | Spain | n = 297 adults (148 females), 42.8 ± 7.8 years | Survey |
| Füzeki et al. [52] | CS | Italy | n = 1500 adults (1126 females), 43.1 ± 11.3 years | Survey |
| Füzéki et al. [53] | CS | Germany | n = 979 adults (703 females), females 42.9 ± 14.0, males 46.7 ± 16.0 years | Survey |
| Gallè et al. [54] | CS | Italy | n = 1430 undergraduate students (937 females), 22.9 ± 3.5 years | Survey |
| Genin et al. [55] | CS | France | n = 15226 children, adolescents, adults, seniors (10353 females), age not reported | Survey |
| Gierc et al. [56] | CS | International | n = 522 adults (452 females), 32.2 ± 13.6 years | Survey |
| Gilic et al. [57] | L | Bosnia and Herzegovina | n = 366 adolescents (322 females), girls: 17.9 ± 1; boys:17 ± 1 years | Survey |
| Giustino et al. [58] | CS | Italy | n = 802 adults (411 females), 32.3 ± 12.8 years | Survey |
| Gjaka et al. [59] | CS | Kosovo | n = 1633 adults (823 females), 24.7 ± 9.3 years | Survey |
| Haider et al. [60] | CS | Austria | n = 652 adults (481 females), 36.0 ± 14.4 years | Survey |
| Han et al. [61] | L | United States | n = 42 hemodialysis patients (9 females), 55 ± 11 years | Survey, Fitbit Charge 2 |
| Hargreaves et al. [62] | L | New Zealand | n = 231 adults (168 females), 42.9 ± 13.8 years | Survey |
| Harrison et al. [63] | CS | United States | n = 9969 adults (5733 females), 62.1 ± 11.2 (>40) years | Survey |
| He et al. [64] | CS | China | n = 339 adults (181 females), male: 36.4 ± 11.9; female: 37.6 ± 12.4 years | Survey, Smartphone Application |
| Hemphill et al. [65] | L | Canada | n = 165 children with congenital heart disease (74 females), 2019: 13 ± 2.3; 2020: 13.2 ± 2.3 years | Fitbit Charge 2 |
| Henriksen et al. [66] | L | Not reported | n = 106 adults (59 females), 40.6 ± 10.6 years | Survey, Wearable activity trackers |
| Hermassi et al. [67] | CS | Qatar | n = 1144 adults (556 females), 33.1 ± 11.1 years | Survey |
| Hermassi et al. [68] | CS | International | n = 1359 handball players (458 females), 22.8 ± 6 years | Survey |
| Huber et al. [69] | CS | Germany | n = 1980 students (1371 females), 23.3 ± 4 years | Survey |
| Huber et al. [70] | CS | Germany | n = 65 older adults (42 females), 73.3 ± 6.0 years | Survey |
| Hume et al. [71] | L | United Kingdom | n = 10 patients with COPD (number of females not reported), age not reported | Accelerometer, Survey |
| Imaz-Aramburu et al. [72] | L | Spain | n = 267 university students (203 females), 20.2 ± 4 years | Survey |
| Jalal et al. [73] | L | Saudi Arabia | n = 628 bachelor students (445 females), 20.5 ± 1.9 years | Survey |
| Janssen et al. [74] | L | United Kingdom | n = 3241 adults (2566 females), 46.2 ± 15.30 years | Survey |
| Jia et al. [75] | CS | China | n = 10082 high school and graduated students (7228 females), 19.8 ± 2.3 years | Survey |
| Joseph et al. [76] | L | United States | n = 589 middle aged and older adults (520 females), 63.2 ± 7.39 years | Survey |
| Karuc et al. [77] | CS | Croatia | n = 91 young adults (59 females), 20-21 years | Survey |
| Kontsevaya et al. [78] | CS | Russia | n = 2432 adults (2104 females), 37.6 ± 13.4 years | Survey |
| Koohsari et al. [79] | L | Japan | n = 2466 adults (1212 females), 39.6 ± 10.7 years | Survey |
| Kouis et al. [80] | L | Cyprus, Greece | n = 108 asthmatic children (45 females), Cyprus: 9.3 ± 1.7; Greece: 9.11 ± 1.8 years | Wearable Sensors |
| Kowalsky et al. [81] | CS | United States | n = 189 college students (138 females), 22.1 ± 4.9 years | Survey |
| LaCaille et al. [82] | L | United States | n = 234 young adults (137 females), 18.1 ± 0.3 years | Survey |
| Lawrence et al. [83] | L | United States | n = 88 students (81 females), 29 ± 10.1 years | Survey |
| Leppä et al. [84] | L | Finland | n = 797 community-dwelling older adults (466 females), 78.7 ± 3.5 years | Survey |
| Li et al. [85] | L | China | n = 19960 adults (2012 females), >18 years | Survey, Huawei mobile phone, smart watch, accelerometers |
| Lopez-Bueno et al. [86] | CS | Spain | n = 2741 adults (1321 females), 34.2 ± 13 years | Survey |
| Lopez-Bueno et al. [87] | CS | Spain | n = 2042 adults (1102 females), 35.9 ± 13.6 years | Survey |
| Lopez-Bueno et al. [88] | CS | Spain | n = 860 children and adolescents (424 females), 9.6 ± 3.6 years | Survey |
| Lopez-Gil et al. [89] | CS | Spain, Brazil | n = 1149 preschoolers, children, adolescents (573 females), Spanish: 12.1 ± 4.6; Brazilian: 10.7 ± 4.3 years | Survey |
| Lopez-Sanchez et al. [90] | CS | Spain | n = 160 adults with chronic conditions (113 females), 18-64 years | Survey |
| Maher et al. [91] | L | United States | n = 107 students (number of females not reported), 21.7 ± 2.6 years | Survey |
| Makizako et al. [92] | CS | Japan | n = 310 older adults (172 females), 71.3 ± 4.4 years | Accelerometer |
| Malanchini et al. [93] | L | Italy | n = 184 patients with implantable cardioverter defibrillators (50 females), 67 ± 14 years | Pacemakers and defibrillators with automatic in-home monitoring system |
| Maltagliati et al. [94] | L | France, Switzerland | n = 113 adults (77 females), 43 ± 18 years | Survey |
| Maltoni et al. [95] | L | Italy | n = 51 adolescent (20 females), 32.6 ± 4.0 years | Survey |
| Marco-Ahulla et al. [96] | L | Spain | n = 20 complete thoracic spinal cord injury full-tile manual wheelchair patients (number of females not reported), 45.4 ± 9.5 years | Survey |
| Martinez-de-Quel et al. [97] | L | Spain | n = 161 adults (60 females), 35 ± 11.2 years | Survey |
| Mascherini et al. [98] | L | Italy | n = 1383 academic population (307 females), female 29.8 ± 12.2  male 33.2 ± 14.5 years | Survey |
| Mason et al. [99] | L | United States | n = 559 (number of females not reported), age not reported | Accelerometers |
| Maugeri et al. [100] | CS | Italy | n = 2524 adults (1426 females), 21-60 years | Survey |
| Mazo et al. [101] | CS | Brazil | n = 111 older adults (56 females), 71.0 ± 6.9 years | Survey |
| McCarthy et al. [102] | L | United Kingdom | n = 5395 adults (3274 females), 41 ± 12 years | Smartphone Application |
| Medrano et al. [103] | L | Spain | n = 106 children and teens (number of females not reported), 12.0 ± 2.6 years | Survey |
| Meyer et al. [104] | CS | United States | n = 3052 adults (1892 females), >18 years | Survey |
| Mirhajianmoghadam et al. [105] | CS | United States | n = 53 non-myopic and myopic children (number of females not reported), 8.3 ± 2.4 (5–12) years | Survey |
| Mishra et al. [106] | L | United States | n = 10 older adults (4 females), 77.3 ± 1.9 years | Wearable Sensor |
| Miyahara et al. [107] | L | Japan | n = 13 elderly people (11 females), 77.5 ± 3.5 years | Accelerometer |
| Mon-Lopez et al. [108] | CS | Spain | n = 120 adults (60 females), 39.7 ± 13.6 years | Survey |
| Moura et al. [109] | L | Brazil | n = 17 older adults (13 females), 65.6 ± 3.8 years | Survey, Accelerometers, cardiorespiratory fitness |
| Mutz-Reimers et al. [110] | CS | Germany | n = 1508 parents and children (768 females), >14 years | Survey |
| Nascimento et al. [111] | L | Brazil | n = 72 older adults (59 females), 67.2 ± 4.9 years | Survey |
| Natalucci et al. [112] | CS | Italy | n = 781 breast cancer patients (781 females), 54.7 ± 8.8 years | Survey |
| Nathan et al. [113] | CS | Australia | n = 157 children (72 females), 6.9 ± 1.7 years | Survey |
| Naughton et al. [114] | L | United Kingdom | n = 1044 adults (758 females), >18 years | Survey |
| Okely et al. [115] | L | International | n = 948 children (465 females), 4.4 ± 0.6 (3-5) years | Survey |
| Oliveira et al. [116] | CS | Brazil | n = 322 adults (223 females), 40 ± 15 (18–89) years | Survey |
| Ong et al. [117] | L | Singapore | n = 1824 adults (941 females), 30.9 ± 4.6 years | Fitbit Ionic, wrist-worn wearable technology |
| Park et al. [118] | L | South Korea | n = 834 adults (381 females), 23.7 ± 6.0 years | Smartphone Application |
| Perez et al. [119] | L | Spain | n = 98 frail older community-dwellers (65 females), 82.4 ± 6.1 years | Survey |
| Pisot et al. [120] | CS | International | n = 4108 adults (2581 females), 32 ± 13.2 years | Survey |
| Pla et al. [121] | L | France | n = 20 athletes (11 females), 17.5 ± 2.4 years | Survey, Heart rate recordings |
| Predieri et al. [122] | L | Italy | n = 62 children and adolescents with diabetes (31 females), T1:11.1 ± 4.4; T2: 11.4 ± 4.4 years | Survey |
| Rastogi et al. [123] | L | India | n = 422 diabetes patients (119 females), age not reported | Survey |
| Rhodes et al. [124] | CS | Canada | n = 1055 adults (536 females), 48.8 ± 16.7 years | Survey |
| Ribeiro de Lima et al. [125] | L | Brazil | n = 34 older adults (34 females), 58.5 ± 6.0 (50-70) years | Survey |
| Richardson et al. [126] | CS | United Kingdom | n = 117 older adults (65 females), male: 76 ± 4; female: 76 ± 4 years | Survey |
| Roberts-Lewis et al. [127] | L | United Kingdom | n = 85 adults with muscle diseases (number of females not reported), age not reported | Accelerometer |
| Rodriguez-Larrad et al. [128] | CS | Spain | n = 13753 students (8967 females), 22.8 ± 5.3 years | Survey |
| Romero-Blanco et al. [129] | CS | Spain | n = 213 students (172 females), 20.5 ± 4.6 years | Survey |
| Rowlands et al. [130] | L | United Kingdom | n = 165 adults with diabetes (74 females), 64.2 ± 8.3 years | Accelerometer |
| Ruiz-Roso et al. [131] | CS | Spain | n = 72 diabetes patients (37 females), years | Survey |
| Sadarangani et al. [132] | CS | Argentina, Chile | n = 1305 adults (1049 females), age not reported | Survey |
| Salman et al. [133] | L | United Kingdom | n = 6219 adults (3445 females), 50–92 years | Survey |
| Sanudo et al. [134] | L | Spain | n = 57 young adults (22 females), 22.5 ± 2.6 years | Survey, Accelerometer |
| Sasaki et al. [135] | CS | Japan | n = 999 older adults (537 females), male: 74.2 ± 6.5; female: 74.7 ± 6.2 years | Survey |
| Sassone et al. [136] | L | Italy | n = 24 patients with implantable cardioverter-defibrillators (7 females), 71 ± 10 years | Accelerometer |
| Savage et al. [137] | L | United Kingdom | n = 214 students (154 females), >18 years | Survey |
| Schlichtiger et al. [138] | CS | Germany | n = 110 older adults (78 females), 66 ± 10 years | Survey |
| Schmidt & Pawlowski [139] | CS | Denmark | n = 1802 teens, younger adults, adults, older adults (1363 females), >14 years | Survey |
| Schmidt et al. [140] | L | Germany | n = 1711 children and adolescents (number of females not reported), 4-17 years | Survey |
| Sekulic et al. [141] | L | Croatia | n = 388 adolescents (126 females), 16.4 ± 1.9 years | Survey |
| Shin et al. [142] | L | South Korea | n = 246 older adults (146 females), 73.8 ± 5.7 years | Survey |
| Sidebottom et al. [143] | CS | United States | n = 291 collage age students (203 females), 18–24 years | Survey |
| Silva Santos et al. [144] | L | Brazil | n = 15 young badminton athletes (9 females), female 18.5 ± 2.6   male 18.7 ± 1.8 years | Accelerometer |
| Silva-Batista et al. [145] | CS | Brazil | n = 478 patients with Parkinson disease (167 females), 67 ± 9.5 years | Survey |
| Song et al. [146] | L | South Korea | n = 100 patients with Parkinson (46 females), 62.3–76.0 years | Survey |
| Souza et al. [147] | CS | Brazil | n = 1368 adults (1094 females), 24–39 years | Survey |
| Srivastav et al. [148] | CS | India | n = 143 physiotherapy professionals and students (number of females not reported), 23–24.8 years | Survey |
| Strutt et al. [149] | CS | Australia | n = 201 community-dwelling older adults (136 females), 70.6 ± 6.5 years | Survey |
| Szabo et al. [150] | CS | Hungary | n = 840 athletes (439 females), >14 years | Survey |
| Theis et al. [151] | CS | United Kingdom | n = 123 children (80 females), 12.3 ± 4.3 years | Survey |
| Tornaghi et al. [152] | CS | Italy | n = 1568 youngsters (number of females not reported), 15-18 years | Survey |
| Ugbolue et al. [153] | L | International | n = 9142 adults (6292 females), female: 41.4 ± 13.1; male: 43.9 ± 14.3 years | Survey |
| van Bakel et al. [154] | L | Netherlands | n = 1433 Dutch cardiovascular disease patients (381 females), 65 (58-70) years | Survey |
| Vetrovsky et al. [155] | L | Czech Republic | n = 3544 heart failure patients (1226 females), 51.6 ± 8.9 years | Accelerometer |
| Wang et al. [156] | L | China | n = 3544 middle aged and older residents (1226 females), ≥40 years | Smartphone Application |
| Wang et al. [157] | CS | Hong Kong | n = 724 (502 females), age not reported | Survey |
| Wang et al. [158] | L | China | n = 1028 adults (502 females), 19-59 years | Survey |
| Wang J et al. [159] | L | Hong Kong | n = 661 (331 females), age not reported | Survey |
| Werneck et al. [160] | CS | Brazil | n = 38353 adults (number of females not reported), age not reported | Survey |
| Wilke et al. [161] | CS | International | n = 13503 adults (7967 females), 39 ± 15 years | Survey |
| Woodruff et al. [162] | L | Canada | n = 121 adults (96 females), 36.2 ± 13.1 years | Activity Trackers |
| Wunsch et al. [163] | L | Germany | n = 961 children and adolescents (447 females), 10.4 ± 4 years | Survey |
| Yamada et al. [164] | CS | Japan | n = 1600 community-dwelling older adults (800 females), 74 ± 5.6 years | Survey |
| Yamada M et al. [165] | L | Japan | n = 937 seniors (458 females), 73.5 ± 5.5 years | Survey |
| Yang et al. [166] | L | China | n = 10081 youths (7228 females), 17.5 ± 1.2 years | Survey |
| Yang et al. [167] | L | United States | n = 430 adults (211 females), 39.1 ± 10.6 years | Smartphone Applications |
| Yang et al. [168] | L | Hong Kong | n = 661 (315 females), age not reported | Survey |
| Zenic et al. [169] | L | Croatia | n = 823 (number of females not reported), 16.5 ± 2.1 years | Survey |
| Zheng et al. [170] | CS | China | n = 631 young adults (386 females), 18-35 years | Survey |
| Zhu et al. [171] | CS | China | n = 1029 adults (711 females), >18 years | Survey |
| Zinner et al. [172] | L | Germany | n = 14 trained kayakers and canoeists (8 females), 17.1 ± 1.9 years | Multi-sensor smartwatch tracking |

CS = cross-sectional, L = longitudinal, PA = Physical Activity

**Suppl. Tab. 3.** Reference list of included studies

1 Abid R, Ammar A, Maaloul R, *et al.* Effect of COVID-19-Related Home Confinement on Sleep Quality, Screen Time and Physical Activity in Tunisian Boys and Girls: A Survey. *IJERPH* 2021;**18**:3065. doi:10.3390/ijerph18063065

2 Ács P, Prémusz V, Morvay-Sey K, *et al.* EFFECTS OF COVID-19 ON PHYSICAL ACTIVITY BEHAVIOR AMONG UNIVERSITY STUDENTS: RESULTS OF A HUNGARIAN ONLINE SURVEY. *hpc* 2020;**14**:174–82. doi:10.5114/hpc.2020.98472

3 Aegerter AM, Deforth M, Sjøgaard G, *et al.* No Evidence for a Decrease in Physical Activity Among Swiss Office Workers During COVID-19: A Longitudinal Study. *Front Psychol* 2021;**12**:620307. doi:10.3389/fpsyg.2021.620307

4 Aguilar-Farias N, Toledo-Vargas M, Miranda-Marquez S, *et al.* Sociodemographic Predictors of Changes in Physical Activity, Screen Time, and Sleep among Toddlers and Preschoolers in Chile during the COVID-19 Pandemic. *IJERPH* 2020;**18**:176. doi:10.3390/ijerph18010176

5 Al-Musharaf S, Aljuraiban G, Bogis R, *et al.* Lifestyle changes associated with COVID-19 quarantine among young Saudi women: A prospective study. *PLoS ONE* 2021;**16**:e0250625. doi:10.1371/journal.pone.0250625

6 Alonso-Martínez AM, Ramírez-Vélez R, García-Alonso Y, *et al.* Physical Activity, Sedentary Behavior, Sleep and Self-Regulation in Spanish Preschoolers during theCOVID-19 Lockdown. *Int J Environ Res Public Health* 2021;:8.

7 Amini H, Isanejad A, Chamani N, *et al.* Physical activity during COVID-19 pandemic in the Iranian population: A brief report. *Heliyon* 2020;**6**:e05411. doi:10.1016/j.heliyon.2020.e05411

8 Ammar A, Brach M, Trabelsi K, *et al.* Effects of COVID-19 Home Confinement on Eating Behaviour and Physical Activity: Results of the ECLB-COVID19 International Online Survey. *Nutrients* 2020;**12**:1583. doi:10.3390/nu12061583

9 Assaloni R, Pellino VC, Puci MV, *et al.* Coronavirus disease (Covid-19): How does the exercise practice in active people with type 1 diabetes change? A preliminary survey. *Diabetes Research and Clinical Practice* 2020;**166**:108297. doi:10.1016/j.diabres.2020.108297

10 Bann D, Villadsen A, Maddock J, *et al.* Changes in the behavioural determinants of health during the COVID-19 pandemic: gender, socioeconomic and ethnic inequalities in five British cohort studies. *J Epidemiol Community Health* 2021;**75**:1136–42. doi:10.1136/jech-2020-215664

11 Barkley JE, Lepp A, Glickman E, *et al.* The Acute Effects of the COVID-19 Pandemic on Physical Activity and Sedentary Behavior in University Students and Employees. 2020;:14.

12 Barone Gibbs B, Kline CE, Huber KA, *et al.* COVID-19 shelter-at-home and work, lifestyle and well-being in desk workers. *Occupational Medicine* 2021;:kqab011. doi:10.1093/occmed/kqab011

13 Bartlett L, Brady JJR, Farrow M, *et al.* Change in modifiable dementia risk factors during COVID‐19 lockdown: The experience of over 50s in Tasmania, Australia. *Alzheimer’s &amp; Dementia: Translational Research &amp; Clinical Interventions* 2021;**7**. doi:10.1002/trc2.12169

14 Beck AL, Huang JC, Lendzion L, *et al.* Impact of the Coronavirus Disease 2019 Pandemic on Parents’ Perception of Health Behaviors in Children With Overweight and Obesity. *Academic Pediatrics* 2021;**21**:1434–40. doi:10.1016/j.acap.2021.05.015

15 Belgen Kaygısız B, Güçhan Topcu Z, Meriç A, *et al.* Determination of exercise habits, physical activity level and anxiety level of postmenopausal women during COVID-19 pandemic. *Health Care for Women International* 2020;**41**:1240–54. doi:10.1080/07399332.2020.1842878

16 Berengüí R, López-Gullón JM, Angosto S. Physical Sports Activities and Exercise Addiction during Lockdown in the Spanish Population. *IJERPH* 2021;**18**:3119. doi:10.3390/ijerph18063119

17 Bertrand L, Shaw KA, Ko J, *et al.* The impact of the coronavirus disease 2019 (COVID-19) pandemic on university students’ dietary intake, physical activity, and sedentary behaviour. *Appl Physiol Nutr Metab* 2021;:1–8. doi:10.1139/apnm-2020-0990

18 Bogataj Jontez N, Novak K, Kenig S, *et al.* The Impact of COVID-19-Related Lockdown on Diet and Serum Markers in Healthy Adults. *Nutrients* 2021;**13**:1082. doi:10.3390/nu13041082

19 Bourdas DI, Zacharakis ED. Impact of COVID-19 Lockdown on Physical Activity in a Sample of Greek Adults. *Sports* 2020;**8**:139. doi:10.3390/sports8100139

20 Branquinho C, Paiva T, Guedes F, *et al.* Health risk behaviors before and during COVID‐19 and gender differences. *J Community Psychol* 2021;:jcop.22705. doi:10.1002/jcop.22705

21 Browne RAV, Macêdo GAD, Cabral LLP, *et al.* Initial impact of the COVID-19 pandemic on physical activity and sedentary behavior in hypertensive older adults: An accelerometer-based analysis. *Experimental Gerontology* 2020;**142**:111121. doi:10.1016/j.exger.2020.111121

22 Buoite Stella A, AjČeviĆ M, Furlanis G, *et al.* Smart technology for physical activity and health assessment during COVID-19 lockdown. *J Sports Med Phys Fitness* 2021;**61**:452–60. doi:10.23736/S0022-4707.20.11373-2

23 Calcaterra V, Iafusco D, Carnevale Pellino V, *et al.* “CoVidentary”: An online exercise training program to reduce sedentary behaviours in children with type 1 diabetes during the COVID-19 pandemic. *Journal of Clinical & Translational Endocrinology* 2021;**25**:100261. doi:10.1016/j.jcte.2021.100261

24 Lorenzo Calvo J, Granado-Peinado M, de la Rubia A, *et al.* Psychological States and Training Habits during the COVID-19 Pandemic Lockdown in Spanish Basketball Athletes. *IJERPH* 2021;**18**:9025. doi:10.3390/ijerph18179025

25 Castañeda-Babarro A, Arbillaga-Etxarri A, Gutiérrez-Santamaría B, *et al.* Physical Activity Change during COVID-19 Confinement. *IJERPH* 2020;**17**:6878. doi:10.3390/ijerph17186878

26 Cheval B, Sivaramakrishnan H, Maltagliati S, *et al.* Relationships between changes in self-reported physical activity, sedentary behaviour and health during the coronavirus (COVID-19) pandemic in France and Switzerland. *Journal of Sports Sciences* 2020;:1–6. doi:10.1080/02640414.2020.1841396

27 Chouchou F, Augustini M, Caderby T, *et al.* The importance of sleep and physical activity on well-being during COVID-19 lockdown: reunion island as a case study. *Sleep Medicine* 2021;**77**:297–301. doi:10.1016/j.sleep.2020.09.014

28 Coughenour C, Gakh M, Pharr JR, *et al.* Changes in Depression and Physical Activity Among College Students on a Diverse Campus After a COVID-19 Stay-at-Home Order. *J Community Health* 2021;**46**:758–66. doi:10.1007/s10900-020-00918-5

29 Curtis RG, Olds T, Ferguson T, *et al.* Changes in diet, activity, weight, and wellbeing of parents during COVID-19 lockdown. *PLoS ONE* 2021;**16**:e0248008. doi:10.1371/journal.pone.0248008

30 Czenczek- Lewandowska E, Wyszyńska J, Leszczak J, *et al.* Health behaviours of young adults during the outbreak of the Covid-19 pandemic – a longitudinal study. *BMC Public Health* 2021;**21**:1038. doi:10.1186/s12889-021-11140-w

31 Davy JP, Scheuermaier K, Roden LC, *et al.* The COVID-19 Lockdown and Changes in Routine-Oriented Lifestyle Behaviors and Symptoms of Depression, Anxiety, and Insomnia in South Africa. *Journal of Physical Activity and Health* 2021;**18**:1046–57. doi:10.1123/jpah.2020-0863

32 de la Vega R, Almendros LJ, Barquín RR, *et al.* Exercise Addiction During the COVID-19 Pandemic: an International Study Confirming the Need for Considering Passion and Perfectionism. *Int J Ment Health Addiction* Published Online First: 3 December 2020. doi:10.1007/s11469-020-00433-7

33 DeJong AF, Fish PN, Hertel J. Running behaviors, motivations, and injury risk during the COVID-19 pandemic: A survey of 1147 runners. *PLOS ONE*;:18.

34 Delisle Nyström C, Alexandrou C, Henström M, *et al.* International Study of Movement Behaviors in the Early Years (SUNRISE): Results from SUNRISE Sweden’s Pilot and COVID-19 Study. *IJERPH* 2020;**17**:8491. doi:10.3390/ijerph17228491

35 Di Sebastiano KM, Chulak-Bozzer T, Vanderloo LM, *et al.* Don’t Walk So Close to Me: Physical Distancing and Adult Physical Activity in Canada. *Front Psychol* 2020;**11**:1895. doi:10.3389/fpsyg.2020.01895

36 Di Stefano V, Battaglia G, Giustino V, *et al.* Significant reduction of physical activity in patients with neuromuscular disease during COVID-19 pandemic: the long-term consequences of quarantine. *J Neurol* 2021;**268**:20–6. doi:10.1007/s00415-020-10064-6

37 Di Stefano V, Ornello R, Gagliardo A, *et al.* Social Distancing in Chronic Migraine during the COVID-19 Outbreak: Results from a Multicenter Observational Study. *Nutrients* 2021;**13**:1361. doi:10.3390/nu13041361

38 Ding D, Cheng M, del Pozo Cruz B, *et al.* How COVID-19 lockdown and reopening affected daily steps: evidence based on 164,630 person-days of prospectively collected data from Shanghai, China. *Int J Behav Nutr Phys Act* 2021;**18**:40. doi:10.1186/s12966-021-01106-x

39 Dobrowolski H, Włodarek D. Body Mass, Physical Activity and Eating Habits Changes during the First COVID-19 Pandemic Lockdown in Poland. *IJERPH* 2021;**18**:5682. doi:10.3390/ijerph18115682

40 Đogaš Z, Lušić Kalcina L, Pavlinac Dodig I, *et al.* The effect of COVID-19 lockdown on lifestyle and mood in Croatian general population: a cross-sectional study. *Croat Med J* 2020;**61**:309–18. doi:10.3325/cmj.2020.61.309

41 Dun Y, Ripley-Gonzalez JW, Zhou N, *et al.* Weight gain in Chinese youth during a 4-month COVID-19 lockdown: a retrospective observational study. *BMJ Open* 2021;**11**:e052451. doi:10.1136/bmjopen-2021-052451

42 Dunton GF, Wang SD, Do B, *et al.* Early effects of the COVID-19 pandemic on physical activity locations and behaviors in adults living in the United States. *Preventive Medicine Reports* 2020;**20**:101241. doi:10.1016/j.pmedr.2020.101241

43 Ellis LA, Lee MD, Ijaz K, *et al.* COVID-19 as ‘Game Changer’ for the Physical Activity and Mental Well-Being of Augmented Reality Game Players During the Pandemic: Mixed Methods Survey Study. *J Med Internet Res* 2020;**22**:e25117. doi:10.2196/25117

44 Elran-Barak R, Mozeikov M. One Month into the Reinforcement of Social Distancing due to the COVID-19 Outbreak: Subjective Health, Health Behaviors, and Loneliness among People with Chronic Medical Conditions. *IJERPH* 2020;**17**:5403. doi:10.3390/ijerph17155403

45 Esain I, Gil SM, Duñabeitia I, *et al.* Effects of COVID-19 Lockdown on Physical Activity and Health-Related Quality of Life in Older Adults Who Regularly Exercise. *Sustainability* 2021;**13**:3771. doi:10.3390/su13073771

46 Fernández-García ÁI, Marin-Puyalto J, Gómez-Cabello A, *et al.* Impact of the Home Confinement Related to COVID-19 on the Device-Assessed Physical Activity and Sedentary Patterns of Spanish Older Adults. *BioMed Research International* 2021;**2021**:1–8. doi:10.1155/2021/5528866

47 Feter N, Caputo EL, Doring IR, *et al.* Sharp increase in depression and anxiety among Brazilian adults during the COVID-19 pandemic: findings from the PAMPA cohort. *Public Health* 2021;**190**:101–7. doi:10.1016/j.puhe.2020.11.013

48 Flanagan EW, Beyl RA, Fearnbach SN, *et al.* The Impact of COVID‐19 Stay‐At‐Home Orders on Health Behaviors in Adults. *Obesity* 2021;**29**:438–45. doi:10.1002/oby.23066

49 Francisco-Pascual J, Rivas-Gándara N, Santos-Ortega A, *et al.* Cardiac biometric variables and arrhythmic events during COVID-19 pandemic lockdown in patients with an implantable cardiac monitor for syncope work-up. *Medicina Clínica* 2021;**156**:496–9. doi:10.1016/j.medcli.2020.12.005

50 Franco I, Bianco A, Bonfiglio C, *et al.* Decreased levels of physical activity: results from a cross-sectional study in southern Italy during the COVID-19 lockdown. *J Sports Med Phys Fitness* 2021;**61**. doi:10.23736/S0022-4707.20.11536-6

51 Franco E, Urosa J, Barakat R, *et al.* Physical Activity and Adherence to the Mediterranean Diet among Spanish Employees in a Health-Promotion Program before and during the COVID-19 Pandemic: The Sanitas-Healthy Cities Challenge. *IJERPH* 2021;**18**:2735. doi:10.3390/ijerph18052735

52 Füzéki E, Schröder J, Carraro N, *et al.* Physical Activity during the First COVID-19-Related Lockdown in Italy. *IJERPH* 2021;**18**:2511. doi:10.3390/ijerph18052511

53 Füzéki E, Schröder J, Groneberg DA, *et al.* Physical Activity and Its Related Factors during the First COVID-19 Lockdown in Germany. *Sustainability* 2021;**13**:5711. doi:10.3390/su13105711

54 Gallè F, Sabella EA, Ferracuti S, *et al.* Sedentary Behaviors and Physical Activity of Italian Undergraduate Students during Lockdown at the Time of CoViD−19 Pandemic. *IJERPH* 2020;**17**:6171. doi:10.3390/ijerph17176171

55 Genin PM, Lambert C, Larras B, *et al.* How Did the COVID-19 Confinement Period Affect Our Physical Activity Level and Sedentary Behaviors? Methodology and First Results From the French National ONAPS Survey. *Journal of Physical Activity and Health* 2021;**18**:296–303. doi:10.1123/jpah.2020-0449

56 Gierc M, Riazi NA, Fagan MJ, *et al.* Strange Days: Adult Physical Activity and Mental Health in the First Two Months of the COVID-19 Pandemic. *Front Public Health* 2021;**9**:567552. doi:10.3389/fpubh.2021.567552

57 Gilic B, Ostojic L, Corluka M, *et al.* Contextualizing Parental/Familial Influence on Physical Activity in Adolescents before and during COVID-19 Pandemic: A Prospective Analysis. *Children* 2020;**7**:125. doi:10.3390/children7090125

58 Giustino V, Parroco AM, Gennaro A, *et al.* Physical Activity Levels and Related Energy Expenditure during COVID-19 Quarantine among the Sicilian Active Population: A Cross-Sectional Online Survey Study. *Sustainability* 2020;**12**:4356. doi:10.3390/su12114356

59 Gjaka M, Feka K, Bianco A, *et al.* The Effect of COVID-19 Lockdown Measures on Physical Activity Levels and Sedentary Behaviour in a Relatively Young Population Living in Kosovo. *JCM* 2021;**10**:763. doi:10.3390/jcm10040763

60 Haider S, Smith L, Markovic L, *et al.* Associations between Physical Activity, Sitting Time, and Time Spent Outdoors with Mental Health during the First COVID-19 Lock Down in Austria. *IJERPH* 2021;**18**:9168. doi:10.3390/ijerph18179168

61 Han M, Preciado P, Thwin O, *et al.* Effect of Statewide Lockdown in Response to COVID-19 Pandemic on Physical Activity Levels of Hemodialysis Patients. *Blood Purif* 2021;**50**:602–9. doi:10.1159/000514935

62 Hargreaves EA, Lee C, Jenkins M, *et al.* Changes in Physical Activity Pre-, During and Post-lockdown COVID-19 Restrictions in New Zealand and the Explanatory Role of Daily Hassles. *Front Psychol* 2021;**12**:642954. doi:10.3389/fpsyg.2021.642954

63 Harrison E, Monroe-Lord L, Carson AD, *et al.* COVID-19 pandemic-related changes in wellness behavior among older Americans. *BMC Public Health* 2021;**21**:755. doi:10.1186/s12889-021-10825-6

64 He M, Xian Y, Lv X, *et al.* Changes in Body Weight, Physical Activity, and Lifestyle During the Semi-lockdown Period After the Outbreak of COVID-19 in China: An Online Survey. *Disaster med public health prep* 2020;:1–6. doi:10.1017/dmp.2020.237

65 Hemphill NM, Kuan MTY, Harris KC. Reduced Physical Activity During COVID-19 Pandemic in Children With Congenital Heart Disease. *Canadian Journal of Cardiology* 2020;**36**:1130–4. doi:10.1016/j.cjca.2020.04.038

66 Henriksen A, Johannessen E, Hartvigsen G, *et al.* Consumer-Based Activity Trackers as a Tool for Physical Activity Monitoring in Epidemiological Studies During the COVID-19 Pandemic: Development and Usability Study. *JMIR Public Health and Surveillance* 2021;**7**:e23806. doi:10.2196/23806

67 Hermassi S, Sellami M, Salman A, *et al.* Effects of COVID-19 Lockdown on Physical Activity, Sedentary Behavior, and Satisfaction with Life in Qatar: A Preliminary Study. *IJERPH* 2021;**18**:3093. doi:10.3390/ijerph18063093

68 Hermassi S, Bouhafs EG, Bragazzi NL, *et al.* Effects of Home Confinement on the Intensity of Physical Activity during the COVID-19 Outbreak in Team Handball According to Country, Gender, Competition Level, and Playing Position: A Worldwide Study. *IJERPH* 2021;**18**:4050. doi:10.3390/ijerph18084050

69 Huber BC, Steffen J, Schlichtiger J, *et al.* Alteration of physical activity during COVID-19 pandemic lockdown in young adults. *J Transl Med* 2020;**18**:410. doi:10.1186/s12967-020-02591-7

70 Huber BC, Schlichtiger J, Drey M, *et al.* Change of the Physical Activity Scale for the Elderly (PASE) Score after COVID-19 Outbreak. ;:8.

71 Hume E, Armstrong M, Manifield J, *et al.* Impact of COVID-19 shielding on physical activity and quality of life in patients with COPD. *Breathe* 2020;**16**:200231. doi:10.1183/20734735.0231-2020

72 Imaz-Aramburu I, Fraile-Bermúdez A-B, Martín-Gamboa BS, *et al.* Influence of the COVID-19 Pandemic on the Lifestyles of Health Sciences University Students in Spain: A Longitudinal Study. *Nutrients* 2021;**13**:1958. doi:10.3390/nu13061958

73 Jalal SM, Beth MRM, Al-Hassan HJM, *et al.* Body Mass Index, Practice of Physical Activity and Lifestyle of Students During COVID-19 Lockdown. *JMDH* 2021;**Volume 14**:1901–10. doi:10.2147/JMDH.S325269

74 Janssen X, Fleming L, Kirk A, *et al.* Changes in Physical Activity, Sitting and Sleep across the COVID-19 National Lockdown Period in Scotland. *Int J Environ Res Public Health* 2020;:10.

75 Jia P, Zhang L, Yu W, *et al.* Impact of COVID-19 lockdown on activity patterns and weight status among youths in China: the COVID-19 Impact on Lifestyle Change Survey (COINLICS). *Int J Obes* 2021;**45**:695–9. doi:10.1038/s41366-020-00710-4

76 Joseph RP, Pituch KA, Guest MA, *et al.* Physical Activity Among Predominantly White Middle-Aged and Older US Adults During the SARS-CoV-2 Pandemic: Results From a National Longitudinal Survey. *Front Public Health* 2021;**9**:652197. doi:10.3389/fpubh.2021.652197

77 Karuc J, Sorić M, Radman I, *et al.* Moderators of Change in Physical Activity Levels during Restrictions Due to COVID-19 Pandemic in Young Urban Adults. *Sustainability* 2020;**12**:6392. doi:10.3390/su12166392

78 Kontsevaya AV, Mukaneeva DK, Myrzamatova AO, *et al.* Changes in physical activity and sleep habits among adults in Russian Federation during COVID-19: a cross-sectional study. *BMC Public Health* 2021;**21**:893. doi:10.1186/s12889-021-10946-y

79 Koohsari MJ, Nakaya T, McCormack GR, *et al.* Changes in Workers’ Sedentary and Physical Activity Behaviors in Response to the COVID-19 Pandemic and Their Relationships With Fatigue: Longitudinal Online Study. *JMIR Public Health Surveill* 2021;**7**:e26293. doi:10.2196/26293

80 Kouis P, Michanikou A, Anagnostopoulou P, *et al.* Use of wearable sensors to assess compliance of asthmatic children in response to lockdown measures for the COVID-19 epidemic. *Sci Rep* 2021;**11**:5895. doi:10.1038/s41598-021-85358-4

81 Kowalsky RJ, Farney TM, Kline CE, *et al.* The impact of the covid-19 pandemic on lifestyle behaviors in U.S. college students. *Journal of American College Health* 2021;:1–6. doi:10.1080/07448481.2021.1923505

82 LaCaille LJ, Hooker SA, Marshall E, *et al.* Change in Perceived Stress and Health Behaviors of Emerging Adults in the Midst of the COVID-19 Pandemic. *Annals of Behavioral Medicine* 2021;**55**:1080–8. doi:10.1093/abm/kaab074

83 Lawrence SA, Garcia J, Stewart C, *et al.* The mental and behavioral health impact of COVID-19 stay at home orders on social work students. *Social Work Education* 2021;:1–15. doi:10.1080/02615479.2021.1883582

84 Leppä H, Karavirta L, Rantalainen T, *et al.* Use of walking modifications, perceived walking difficulty and changes in outdoor mobility among community-dwelling older people during COVID-19 restrictions. *Aging Clin Exp Res* 2021;**33**:2909–16. doi:10.1007/s40520-021-01956-2

85 Li J-W, Guo Y-T, Di Tanna GL, *et al.* Vital Signs During the COVID-19 Outbreak: A Retrospective Analysis of 19,960 Participants in Wuhan and Four Nearby Capital Cities in China. *gh* 2021;**16**:47. doi:10.5334/gh.913

86 López-Bueno R, Calatayud J, Casaña J, *et al.* COVID-19 Confinement and Health Risk Behaviors in Spain. *Front Psychol* 2020;**11**:1426. doi:10.3389/fpsyg.2020.01426

87 López-Bueno R, Calatayud J, Andersen LL, *et al.* Immediate Impact of the COVID-19 Confinement on Physical Activity Levels in Spanish Adults. *Sustainability* 2020;**12**:5708. doi:10.3390/su12145708

88 López-Bueno R, López-Sánchez GF, Casajús JA, *et al.* Health-Related Behaviors Among School-Aged Children and Adolescents During the Spanish Covid-19 Confinement. *Front Pediatr* 2020;**8**:573. doi:10.3389/fped.2020.00573

89 López-Gil JF, Tremblay MS, Brazo-Sayavera J. Changes in Healthy Behaviors and Meeting 24-h Movement Guidelines in Spanish and Brazilian Preschoolers, Children and Adolescents during the COVID-19 Lockdown. *Children* 2021;**8**:83. doi:10.3390/children8020083

90 López-Sánchez GF, López-Bueno R, Gil-Salmerón A, *et al.* Comparison of physical activity levels in Spanish adults with chronic conditions before and during COVID-19 quarantine. *European Journal of Public Health* 2021;**31**:161–6. doi:10.1093/eurpub/ckaa159

91 Maher JP, Hevel DJ, Reifsteck EJ, *et al.* Physical activity is positively associated with college students’ positive affect regardless of stressful life events during the COVID-19 pandemic. *Psychology of Sport and Exercise* 2021;**52**:101826. doi:10.1016/j.psychsport.2020.101826

92 Makizako H, Liu-Ambrose T, Shimada H, *et al.* Moderate-Intensity Physical Activity, Hippocampal Volume, and Memory in Older Adults With Mild Cognitive Impairment. *The Journals of Gerontology Series A: Biological Sciences and Medical Sciences* 2015;**70**:480–6. doi:10.1093/gerona/glu136

93 Malanchini G, Malacrida M, Ferrari P, *et al.* Impact of the Coronavirus Disease-19 Outbreak on Physical Activity of Patients With Implantable Cardioverter Defibrillators. *Journal of Cardiac Failure* 2020;**26**:898–9. doi:10.1016/j.cardfail.2020.08.005

94 Maltagliati S, Rebar A, Fessler L, *et al.* Evolution of physical activity habits after a context change: The case of COVID‐19 lockdown. *Br J Health Psychol* 2021;**26**:1135–54. doi:10.1111/bjhp.12524

95 Maltoni G, Zioutas M, Deiana G, *et al.* Gender differences in weight gain during lockdown due to COVID-19 pandemic in adolescents with obesity. *Nutrition, Metabolism and Cardiovascular Diseases* 2021;**31**:2181–5. doi:10.1016/j.numecd.2021.03.018

96 Marco-Ahulló A, Montesinos-Magraner L, González L-M, *et al.* Impact of COVID-19 on the self-reported physical activity of people with complete thoracic spinal cord injury full-time manual wheelchair users. *The Journal of Spinal Cord Medicine* 2021;:1–5. doi:10.1080/10790268.2020.1857490

97 Martinez-de-Quel O, Suarez-Iglesias D, Lopez-Flores M, *et al.* Physical activity, dietary habits and sleep quality before and during COVID-19 lockdown: A longitudinal study. *Appetite* 2021;**158**:105019. doi:10.1016/j.appet.2020.105019

98 Mascherini G, Catelan D, Pellegrini-Giampietro DE, *et al.* Changes in physical activity levels, eating habits and psychological well-being during the Italian COVID-19 pandemic lockdown: Impact of socio-demographic factors on the Florentine academic population. *PLoS ONE* 2021;**16**:e0252395. doi:10.1371/journal.pone.0252395

99 Mason MR, Hudgins JH, Campbell MS, *et al.* Changes in physical activity during the initial stages of the COVID-19 pandemic. *Journal of Sports Sciences* 2021;:1–9. doi:10.1080/02640414.2021.1976569

100 Maugeri G, Castrogiovanni P, Battaglia G, *et al.* The impact of physical activity on psychological health during Covid-19 pandemic in Italy. *Heliyon* 2020;**6**:e04315. doi:10.1016/j.heliyon.2020.e04315

101 Mazo GZ, Fank F, Franco PS, *et al.* Impact of Social Isolation on Physical Activity and Factors Associated With Sedentary Behavior in Older Adults During the COVID-19 Pandemic. *Journal of Aging and Physical Activity* 2022;**30**:148–52. doi:10.1123/japa.2020-0456

102 McCarthy H, Potts HWW, Fisher A. Physical Activity Behavior Before, During, and After COVID-19 Restrictions: Longitudinal Smartphone-Tracking Study of Adults in the United Kingdom. *J Med Internet Res* 2021;**23**:e23701. doi:10.2196/23701

103 Medrano M, Cadenas‐Sanchez C, Oses M, *et al.* Changes in lifestyle behaviours during the COVID ‐19 confinement in Spanish children: A longitudinal analysis from the MUGI project. *Pediatric Obesity* Published Online First: 24 September 2020. doi:10.1111/ijpo.12731

104 Meyer J, McDowell C, Lansing J, *et al.* Changes in Physical Activity and Sedentary Behavior in Response to COVID-19 and Their Associations with Mental Health in 3052 US Adults. *IJERPH* 2020;**17**:6469. doi:10.3390/ijerph17186469

105 Mirhajianmoghadam H, Piña A, Ostrin LA. Objective and Subjective Behavioral Measures in Myopic and Non-Myopic Children During the COVID-19 Pandemic. *Trans Vis Sci Tech* 2021;**10**:4. doi:10.1167/tvst.10.11.4

106 Mishra R, Park C, York MK, *et al.* Decrease in Mobility during the COVID-19 Pandemic and Its Association with Increase in Depression among Older Adults: A Longitudinal Remote Mobility Monitoring Using a Wearable Sensor. *Sensors* 2021;**21**:3090. doi:10.3390/s21093090

107 Miyahara S, Tanikawa Y, Hirai H, *et al.* Impact of the state of emergency enacted due to the COVID-19 pandemic on the physical activity of the elderly in Japan. *J Phys Ther Sci* 2021;**33**:345–50. doi:10.1589/jpts.33.345

108 Mon-López D, Bernardez-Vilaboa R, Fernandez-Balbuena AA, *et al.* The Influence of COVID-19 Isolation on Physical Activity Habits and Its Relationship with Convergence Insufficiency. *IJERPH* 2020;**17**:7406. doi:10.3390/ijerph17207406

109 Moura EF, Cabral DAR, Rêgo MLM, *et al.* Associations of objectively measured movement behavior and cardiorespiratory fitness with mental health and quality of life in older adults with hypertension: an exploratory analysis during the COVID-19 pandemic. *Aging & Mental Health* 2021;:1–8. doi:10.1080/13607863.2021.1942436

110 Mutz M, Reimers AK. Leisure time sports and exercise activities during the COVID-19 pandemic: a survey of working parents. *Ger J Exerc Sport Res* 2021;**51**:384–9. doi:10.1007/s12662-021-00730-w

111 Nascimento RJ do, Barbosa Filho VC, Rech CR, *et al.* Changes in Health-Related Quality of Life and Physical Activity Among Older Adults in the First-Wave COVID-19 Outbreak: A Longitudinal Analysis. *Journal of Aging and Physical Activity* 2021;:1–8. doi:10.1123/japa.2021-0104

112 Natalucci V, Villarini M, Emili R, *et al.* Special Attention to Physical Activity in Breast Cancer Patients during the First Wave of COVID-19 Pandemic in Italy: The DianaWeb Cohort. *JPM* 2021;**11**:381. doi:10.3390/jpm11050381

113 Nathan A, George P, Ng M, *et al.* Impact of COVID-19 Restrictions on Western Australian Children’s Physical Activity and Screen Time. *IJERPH* 2021;**18**:2583. doi:10.3390/ijerph18052583

114 Naughton F, Ward E, Khondoker M, *et al.* Health behaviour change during the UK COVID‐19 lockdown: Findings from the first wave of the C‐19 health behaviour and well‐being daily tracker study. *Br J Health Psychol* 2021;:bjhp.12500. doi:10.1111/bjhp.12500

115 Okely AD, Kariippanon KE, Guan H, *et al.* Global effect of COVID-19 pandemic on physical activity, sedentary behaviour and sleep among 3- to 5-year-old children: a longitudinal study of 14 countries. *BMC Public Health* 2021;**21**:940. doi:10.1186/s12889-021-10852-3

116 Oliveira GF, Marin TC, Apolinário N, *et al.* Association of morningness–eveningness preference with physical activity during the COVID-19 pandemic social distancing: a cross-sectional survey in Brazil: Circadian rhythm, physical activity and isolation. *Chronobiology International* 2021;**38**:1432–40. doi:10.1080/07420528.2021.1931276

117 Ong JL, Lau T, Massar SAA, *et al.* COVID-19-related mobility reduction: heterogenous effects on sleep and physical activity rhythms. *Sleep* 2021;**44**:zsaa179. doi:10.1093/sleep/zsaa179

118 Park J-H, Yoo E, Kim Y, *et al.* What Happened Pre- and during COVID-19 in South Korea? Comparing Physical Activity, Sleep Time, and Body Weight Status. *IJERPH* 2021;**18**:5863. doi:10.3390/ijerph18115863

119 Perez LM, Castellano-Tejedor C, Cesari M, *et al.* Depressive Symptoms, Fatigue and Social Relationships Influenced Physical Activity in Frail Older Community-Dwellers during the Spanish Lockdown due to the COVID-19 Pandemic. *International Journal of Environmental Research and Public Health* 2021;**18**:808. doi:10.3390/ijerph18020808

120 Pišot S, Milovanović I, Šimunič B, *et al.* Maintaining everyday life praxis in the time of COVID-19 pandemic measures (ELP-COVID-19 survey). *European Journal of Public Health* 2020;**30**:1181–6. doi:10.1093/eurpub/ckaa157

121 Pla R, Bosquet L, Aubry A, *et al.* Resting Heart Rate Measurement in Elite Athletes during COVID-19 Lockdown: The Impact of Decreased Physical Activity. *Sustainability* 2021;**13**:2970. doi:10.3390/su13052970

122 Predieri B, Leo F, Candia F, *et al.* Glycemic Control Improvement in Italian Children and Adolescents With Type 1 Diabetes Followed Through Telemedicine During Lockdown Due to the COVID-19 Pandemic. *Front Endocrinol* 2020;**11**:595735. doi:10.3389/fendo.2020.595735

123 Rastogi A, Hiteshi P, Bhansali A. Improved glycemic control amongst people with long-standing diabetes during COVID-19 lockdown: a prospective, observational, nested cohort study. *Int J Diabetes Dev Ctries* 2020;**40**:476–81. doi:10.1007/s13410-020-00880-x

124 Rhodes RE, Liu S, Lithopoulos A, *et al.* Correlates of Perceived Physical Activity Transitions during the COVID‐19 Pandemic among Canadian Adults. *Appl Psychol Health Well‐Being* 2020;**12**:1157–82. doi:10.1111/aphw.12236

125 Ribeiro de Lima JG, Abud GF, Freitas EC de, *et al.* Effects of the COVID-19 pandemic on the global health of women aged 50 to 70 years. *Experimental Gerontology* 2021;**150**:111349. doi:10.1016/j.exger.2021.111349

126 Richardson DL, Duncan MJ, Clarke ND, *et al.* The influence of COVID-19 measures in the United Kingdom on physical activity levels, perceived physical function and mood in older adults: A survey-based observational study. *Journal of Sports Sciences* 2020;:1–13. doi:10.1080/02640414.2020.1850984

127 Roberts-Lewis SF, Ashworth M, White CM, *et al.* COVID-19 lockdown impact on the physical activity of adults with progressive muscle diseases. *BMJ Neurol Open* 2021;**3**:e000140. doi:10.1136/bmjno-2021-000140

128 Rodríguez-Larrad A, Mañas A, Labayen I, *et al.* Impact of COVID-19 Confinement on Physical Activity and Sedentary Behaviour in Spanish University Students: Role of Gender. *IJERPH* 2021;**18**:369. doi:10.3390/ijerph18020369

129 Romero-Blanco C, Rodríguez-Almagro J, Onieva-Zafra MD, *et al.* Physical Activity and Sedentary Lifestyle in University Students: Changes during Confinement Due to the COVID-19 Pandemic. *IJERPH* 2020;**17**:6567. doi:10.3390/ijerph17186567

130 Rowlands AV, Henson JJ, Coull NA, *et al.* The impact of COVID‐19 restrictions on accelerometer‐assessed physical activity and sleep in individuals with type 2 diabetes. *Diabet Med* 2021;**38**. doi:10.1111/dme.14549

131 Ruiz-Roso MB, Knott-Torcal C, Matilla-Escalante DC, *et al.* COVID-19 Lockdown and Changes of the Dietary Pattern and Physical Activity Habits in a Cohort of Patients with Type 2 Diabetes Mellitus. *Nutrients* 2020;**12**:2327. doi:10.3390/nu12082327

132 Sadarangani KP, De Roia GF, Lobo P, *et al.* Changes in Sitting Time, Screen Exposure and Physical Activity during COVID-19 Lockdown in South American Adults: A Cross-Sectional Study. *IJERPH* 2021;**18**:5239. doi:10.3390/ijerph18105239

133 Salman D, Beaney T, E Robb C, *et al.* Impact of social restrictions during the COVID-19 pandemic on the physical activity levels of adults aged 50–92 years: a baseline survey of the CHARIOT COVID-19 Rapid Response prospective cohort study. *BMJ Open* 2021;**11**:e050680. doi:10.1136/bmjopen-2021-050680

134 Sañudo B, Fennell C, Sánchez-Oliver AJ. Objectively-Assessed Physical Activity, Sedentary Behavior, Smartphone Use, and Sleep Patterns Pre- and during-COVID-19 Quarantine in Young Adults from Spain. *Sustainability* 2020;**12**:5890. doi:10.3390/su12155890

135 Sasaki S, Sato A, Tanabe Y, *et al.* Associations between Socioeconomic Status, Social Participation, and Physical Activity in Older People during the COVID-19 Pandemic: A Cross-Sectional Study in a Northern Japanese City. *IJERPH* 2021;**18**:1477. doi:10.3390/ijerph18041477

136 Sassone B, Mandini S, Grazzi G, *et al.* Impact of COVID-19 Pandemic on Physical Activity in Patients With Implantable Cardioverter-Defibrillators. *Journal of Cardiopulmonary Rehabilitation and Prevention* 2020;**40**:285–6. doi:10.1097/HCR.0000000000000539

137 Savage MJ, James R, Magistro D, *et al.* Mental health and movement behaviour during the COVID-19 pandemic in UK university students: Prospective cohort study. *Mental Health and Physical Activity* 2020;**19**:100357. doi:10.1016/j.mhpa.2020.100357

138 Schlichtiger J, Steffen J, Huber BC, *et al.* Physical activity during COVID-19 lockdown in older adults. *J Sports Med Phys Fitness* 2020;**61**. doi:10.23736/S0022-4707.20.11726-2

139 Schmidt T, Pawlowski CS. Physical Activity in Crisis: The Impact of COVID-19 on Danes’ Physical Activity Behavior. *Front Sports Act Living* 2021;**2**:610255. doi:10.3389/fspor.2020.610255

140 Schmidt SCE, Anedda B, Burchartz A, *et al.* Physical activity and screen time of children and adolescents before and during the COVID-19 lockdown in Germany: a natural experiment. *Sci Rep* 2020;**10**:21780. doi:10.1038/s41598-020-78438-4

141 Sekulic D, Blazevic M, Gilic B, *et al.* Prospective Analysis of Levels and Correlates of Physical Activity during COVID-19 Pandemic and Imposed Rules of Social Distancing; Gender Specific Study among Adolescents from Southern Croatia. *Sustainability* 2020;**12**:4072. doi:10.3390/su12104072

142 Shin SM, Oh TJ, Choi SH, *et al.* Effects of Social Distancing on Diabetes Management in Older Adults during COVID-19 Pandemic. *Diabetes Metab J* 2021;**45**:765–72. doi:10.4093/dmj.2021.0096

143 Sidebottom C, Ullevig S, Cheever K, *et al.* Effects of COVID-19 pandemic and quarantine period on physical activity and dietary habits of college-aged students. *Sports Medicine and Health Science* 2021;**3**:228–35. doi:10.1016/j.smhs.2021.08.005

144 da Silva Santos AM, Rossi FE, dos Santos Nunes de Moura HP, *et al.* COVID-19 pandemic impacts physical activity levels and sedentary time but not sleep quality in young badminton athletes. *Sport Sci Health* 2021;**17**:969–77. doi:10.1007/s11332-021-00763-6

145 Silva-Batista C, Coelho DB, Júnior RCF, *et al.* Multidimensional Factors Can Explain the Clinical Worsening in People With Parkinson’s Disease During the COVID-19 Pandemic: A Multicenter Cross-Sectional Trial. *Front Neurol* 2021;**12**:708433. doi:10.3389/fneur.2021.708433

146 Song J, Ahn JH, Choi I, *et al.* The changes of exercise pattern and clinical symptoms in patients with Parkinson’s disease in the era of COVID-19 pandemic. *Parkinsonism & Related Disorders* 2020;**80**:148–51. doi:10.1016/j.parkreldis.2020.09.034

147 Souza TC, Oliveira LA, Daniel MM, *et al.* Lifestyle and eating habits before and during COVID-19 quarantine in Brazil. *Public Health Nutr* 2021;:1–11. doi:10.1017/S136898002100255X

148 Srivastav AK, Sharma N, Samuel AJ. Impact of Coronavirus disease-19 (COVID-19) lockdown on physical activity and energy expenditure among physiotherapy professionals and students using web-based open E-survey sent through WhatsApp, Facebook and Instagram messengers. *Clinical Epidemiology and Global Health* 2021;**9**:78–84. doi:10.1016/j.cegh.2020.07.003

149 Strutt PA, Johnco CJ, Chen J, *et al.* Stress and Coping in Older Australians During COVID-19: Health, Service Utilization, Grandparenting, and Technology Use. *Clinical Gerontologist* 2021;:1–13. doi:10.1080/07317115.2021.1884158

150 Szabo A, Ábrahám J. The psychological benefits of recreational running: A field study. *Psychology, Health & Medicine* 2013;**18**:251–61. doi:10.1080/13548506.2012.701755

151 Theis N, Campbell N, De Leeuw J, *et al.* The effects of COVID-19 restrictions on physical activity and mental health of children and young adults with physical and/or intellectual disabilities. *Disability and Health Journal* 2021;:101064. doi:10.1016/j.dhjo.2021.101064

152 Tornaghi M, Lovecchio N, Vandoni M, *et al.* Physical activity levels across COVID-19 outbreak in youngsters of Northwestern Lombardy. ;:20.

153 Ugbolue U, Duclos M, Urzeala C, *et al.* An Assessment of the Novel COVISTRESS Questionnaire: COVID-19 Impact on Physical Activity, Sedentary Action and Psychological Emotion. *JCM* 2020;**9**:3352. doi:10.3390/jcm9103352

154 van Bakel BMA, Bakker EA, de Vries F, *et al.* Impact of COVID-19 lockdown on physical activity and sedentary behaviour in Dutch cardiovascular disease patients. *Neth Heart J* 2021;**29**:273–9. doi:10.1007/s12471-021-01550-1

155 Vetrovsky T, Frybova T, Gant I, *et al.* The detrimental effect of COVID‐19 nationwide quarantine on accelerometer‐assessed physical activity of heart failure patients. *ESC Heart Failure* 2020;**7**:2093–7. doi:10.1002/ehf2.12916

156 Wang Y, Zhang Y, Bennell K, *et al.* Physical Distancing Measures and Walking Activity in Middle-aged and Older Residents in Changsha, China, During the COVID-19 Epidemic Period: Longitudinal Observational Study. *J Med Internet Res* 2020;**22**:e21632. doi:10.2196/21632

157 Wang J, Yeoh EK, Yung TKC, *et al.* Change in eating habits and physical activities before and during the COVID-19 pandemic in Hong Kong: a cross‐sectional study via random telephone survey. *J Int Soc Sports Nutr* 2021;**18**:33. doi:10.1186/s12970-021-00431-7

158 Wang H, Feng L, Zhang Y, *et al.* Changes in Chinese Adults’ Physical Activity Behavior and Determinants before and during the COVID-19 Pandemic. *JCM* 2021;**10**:3069. doi:10.3390/jcm10143069

159 Wang J, Yang Y, Peng J, *et al.* Moderation effect of urban density on changes in physical activity during the coronavirus disease 2019 pandemic. *Sustainable Cities and Society* 2021;**72**:103058. doi:10.1016/j.scs.2021.103058

160 Werneck AO, Silva DR, Malta DC, *et al.* Changes in the clustering of unhealthy movement behaviors during the COVID-19 quarantine and the association with mental health indicators among Brazilian adults. *Translational Behavioral Medicine* 2020;:ibaa095. doi:10.1093/tbm/ibaa095

161 Wilke. A Pandemic within the Pandemic? Physical Activity Levels Substantially Decreased in Countries Affected by COVID-19. *International Journal of Environmental Research and Public Health*

162 Woodruff SJ, Coyne P, St‐Pierre E. Stress, physical activity, and screen‐related sedentary behaviour within the first month of the COVID‐19 pandemic. *Appl Psychol Health Well‐Being* 2021;**13**:454–68. doi:10.1111/aphw.12261

163 Wunsch K, Nigg C, Niessner C, *et al.* The Impact of COVID-19 on the Interrelation of Physical Activity, Screen Time and Health-Related Quality of Life in Children and Adolescents in Germany: Results of the Motorik-Modul Study. *Children* 2021;**8**:98. doi:10.3390/children8020098

164 Yamada M, Kimura Y, Ishiyama D, *et al.* Effect of the COVID-19 Epidemic on Physical Activity in Community-Dwelling Older Adults in Japan: A Cross-Sectional Online Survey. *J Nutr Health Aging* 2020;**24**:948–50. doi:10.1007/s12603-020-1501-6

165 Yamada M, Kimura Y, Ishiyama D, *et al.* The Influence of the COVID-19 Pandemic on Physical Activity and New Incidence of Frailty among Initially Non-Frail Older Adults in Japan: A Follow-Up Online Survey. *J Nutr Health Aging* 2021;**25**:751–6. doi:10.1007/s12603-021-1634-2

166 Yang S, Guo B, Ao L, *et al.* Obesity and activity patterns before and during COVID ‐19 lockdown among youths in China. *Clin Obes* 2020;**10**. doi:10.1111/cob.12416

167 Yang Y, Koenigstorfer J. Determinants of physical activity maintenance during the Covid-19 pandemic: a focus on fitness apps. *Translational Behavioral Medicine* 2020;**10**:835–42. doi:10.1093/tbm/ibaa086

168 Yang Y, Lu Y, Yang L, *et al.* Urban greenery cushions the decrease in leisure-time physical activity during the COVID-19 pandemic: A natural experimental study. *Urban Forestry & Urban Greening* 2021;**62**:127136. doi:10.1016/j.ufug.2021.127136

169 Zenic N, Taiar R, Gilic B, *et al.* Levels and Changes of Physical Activity in Adolescents during the COVID-19 Pandemic: Contextualizing Urban vs. Rural Living Environment. *Applied Sciences* 2020;**10**:3997. doi:10.3390/app10113997

170 Zheng C, Huang WY, Sheridan S, *et al.* COVID-19 Pandemic Brings a Sedentary Lifestyle in Young Adults: A Cross-Sectional and Longitudinal Study. *IJERPH* 2020;**17**:6035. doi:10.3390/ijerph17176035

171 Zhu Y, Wang Z, Maruyama H, *et al.* Effect of the COVID-19 lockdown period on the physical condition, living habits, and physical activity of citizens in Beijing, China. *J Phys Ther Sci* 2021;**33**:632–6. doi:10.1589/jpts.33.632

172 Zinner C, Matzka M, Leppich R, *et al.* The Impact of the German Strategy for Containment of Coronavirus SARS-CoV-2 on Training Characteristics, Physical Activity and Sleep of Highly Trained Kayakers and Canoeists: A Retrospective Observational Study. *Front Sports Act Living* 2020;**2**:579830. doi:10.3389/fspor.2020.579830

**Suppl. Tab. 4.** Ratings of study risk of bias (adapted Downs & Black checklist)

| **Study** | **Reporting** | | | | | | | | **External validity** | **Internal validity**  **(Bias)** | | | | **Internal validity (confounding)** | | **Power** | **Total score** |
| --- | --- | --- | --- | --- | --- | --- | --- | --- | --- | --- | --- | --- | --- | --- | --- | --- | --- |
|  | Objective | Outcomes | Sample | Confounders | Results | Variability estimates | Follow-up | Probability values | Representative sample | Data dregding | Time periods | Appropriate statistics | Valid/reliable measures | Sampling method | Confolounder adjustment | Adequate sample size |  |
| Abid et al. [1] | 1 | 1 | 0 | 0 | 1 | 0 | 1 | 1 | 1 | 1 | 1 | 1 | 1 | 1 | 1 | 1 | 13 |
| Acs et al. [2] | 1 | 1 | 1 | 1 | 1 | 1 | 0 | 1 | 0 | 1 | 0 | 1 | 1 | 1 | 1 | 1 | 13 |
| Aegerter et al. [3] | 1 | 1 | 1 | 1 | 1 | 1 | 0 | 0 | 0 | 0 | 1 | 1 | 1 | 1 | 0 | 0 | 10 |
| Aguilar-Farias et al. [4] | 1 | 1 | 1 | 1 | 1 | 1 | 1 | 1 | 0 | 1 | 1 | 1 | 1 | 1 | 1 | 1 | 15 |
| Al-Musharaf et al. [5] | 0 | 1 | 1 | 1 | 1 | 1 | 1 | 1 | 1 | 1 | 1 | 1 | 1 | 1 | 1 | 0 | 14 |
| Alonso-Martinez et al. [6] | 1 | 1 | 1 | 1 | 1 | 1 | 0 | 1 | 0 | 1 | 0 | 1 | 1 | 1 | 1 | 1 | 13 |
| Amini et al. [7] | 1 | 1 | 1 | 1 | 1 | 1 | 0 | 1 | 0 | 1 | 0 | 1 | 1 | 1 | 1 | 1 | 13 |
| Ammar et al. [8] | 1 | 1 | 1 | 1 | 1 | 1 | 0 | 1 | 0 | 1 | 0 | 1 | 1 | 1 | 1 | 1 | 13 |
| Assaloni et al. [9] | 1 | 1 | 1 | 1 | 1 | 1 | 0 | 1 | 0 | 1 | 1 | 1 | 1 | 0 | 1 | 1 | 13 |
| Bann et al. [10] | 1 | 1 | 1 | 1 | 1 | 1 | 1 | 1 | 1 | 1 | 1 | 1 | 1 | 1 | 1 | 1 | 16 |
| Barkley et al. [11] | 1 | 1 | 1 | 1 | 1 | 1 | 1 | 1 | 0 | 1 | 0 | 1 | 1 | 1 | 1 | 1 | 14 |
| Barone Gibbs et al. [12] | 1 | 1 | 1 | 1 | 1 | 1 | 1 | 1 | 0 | 1 | 1 | 0 | 1 | 1 | 0 | 0 | 12 |
| Bartlett et al. [13] | 1 | 1 | 1 | 1 | 1 | 1 | 0 | 1 | 0 | 1 | 1 | 1 | 1 | 0 | 1 | 0 | 12 |
| Beck et al. [14] | 1 | 1 | 1 | 1 | 1 | 1 | 1 | 0 | 0 | 1 | 1 | 1 | 1 | 1 | 0 | 1 | 13 |
| Belgen et al. [15] | 1 | 1 | 1 | 1 | 1 | 1 | 0 | 1 | 0 | 1 | 0 | 1 | 1 | 1 | 1 | 1 | 13 |
| Berengüi et al. [16] | 1 | 1 | 1 | 1 | 1 | 1 | 1 | 1 | 0 | 1 | 1 | 1 | 1 | 1 | 0 | 0 | 13 |
| Bertrand et al. [17] | 1 | 1 | 1 | 1 | 1 | 1 | 0 | 1 | 0 | 1 | 1 | 1 | 1 | 1 | 1 | 1 | 14 |
| Bogataj Jontez et al. [18] | 1 | 1 | 1 | 1 | 1 | 1 | 1 | 1 | 0 | 1 | 1 | 1 | 1 | 1 | 0 | 1 | 14 |
| Bourdas et al. [19] | 1 | 1 | 1 | 1 | 1 | 1 | 0 | 0 | 0 | 1 | 0 | 1 | 1 | 0 | 1 | 1 | 11 |
| Branquinho et al. [20] | 1 | 0 | 1 | 1 | 1 | 1 | 1 | 1 | 0 | 1 | 0 | 1 | 0 | 0 | 0 | 0 | 9 |
| Browne et al. [21] | 1 | 1 | 1 | 1 | 1 | 1 | 1 | 1 | 0 | 1 | 0 | 1 | 1 | 1 | 1 | 1 | 14 |
| Buoite Stella et al. [22] | 1 | 1 | 1 | 1 | 1 | 1 | 1 | 1 | 0 | 1 | 0 | 1 | 1 | 1 | 1 | 1 | 14 |
| Calcaterra et al. [23] | 1 | 1 | 1 | 1 | 1 | 1 | 1 | 1 | 0 | 1 | 1 | 1 | 1 | 1 | 1 | 1 | 15 |
| Calvo et al. [24] | 1 | 1 | 1 | 1 | 1 | 1 | 1 | 1 | 1 | 1 | 1 | 1 | 1 | 1 | 1 | 1 | 16 |
| Castaneda-Babarro et al. [25] | 1 | 1 | 1 | 1 | 1 | 1 | 0 | 1 | 0 | 1 | 1 | 1 | 1 | 1 | 1 | 1 | 14 |
| Cheval et al. [26] | 1 | 1 | 1 | 1 | 1 | 1 | 0 | 1 | 0 | 1 | 1 | 1 | 1 | 1 | 0 | 0 | 12 |
| Chouchou et al. [27] | 1 | 1 | 1 | 1 | 1 | 1 | 0 | 1 | 0 | 1 | 0 | 1 | 1 | 1 | 1 | 1 | 13 |
| Coughenour et al. [28] | 1 | 1 | 1 | 1 | 1 | 1 | 1 | 1 | 0 | 1 | 1 | 1 | 1 | 1 | 1 | 0 | 14 |
| Curtis et al. [29] | 1 | 1 | 1 | 1 | 1 | 1 | 1 | 1 | 1 | 1 | 1 | 1 | 1 | 1 | 0 | 1 | 15 |
| Czenczek- Lewandowska et al. [30] | 1 | 1 | 1 | 1 | 1 | 1 | 1 | 1 | 0 | 1 | 1 | 1 | 1 | 1 | 0 | 0 | 13 |
| Davy et al. [31] | 1 | 1 | 1 | 1 | 1 | 1 | 1 | 1 | 0 | 1 | 1 | 1 | 1 | 1 | 1 | 1 | 15 |
| de la Vega et al. [32] | 1 | 1 | 1 | 1 | 1 | 1 | 0 | 1 | 0 | 1 | 0 | 1 | 1 | 0 | 1 | 1 | 12 |
| DeJong et al. [33] | 1 | 1 | 1 | 1 | 1 | 1 | 0 | 1 | 1 | 1 | 0 | 1 | 0 | 1 | 1 | 1 | 13 |
| Delisle Nystrom et al. [34] | 1 | 1 | 1 | 1 | 1 | 1 | 1 | 1 | 0 | 1 | 0 | 1 | 1 | 1 | 1 | 1 | 14 |
| Di Sebastiano et al. [35] | 1 | 1 | 1 | 1 | 0 | 0 | 0 | 1 | 0 | 1 | 0 | 1 | 1 | 1 | 1 | 1 | 11 |
| Di Stefano et al. [36] | 1 | 1 | 0 | 1 | 1 | 1 | 0 | 1 | 0 | 1 | 1 | 1 | 1 | 1 | 1 | 1 | 13 |
| Di Stefano et al. [37] | 1 | 1 | 1 | 1 | 1 | 0 | 0 | 1 | 0 | 1 | 1 | 1 | 1 | 1 | 0 | 1 | 12 |
| Ding et al. [38] | 1 | 1 | 1 | 1 | 1 | 1 | 1 | 1 | 1 | 1 | 1 | 1 | 1 | 1 | 0 | 0 | 14 |
| Dobrowolski et al. [39] | 1 | 1 | 1 | 1 | 1 | 1 | 1 | 0 | 1 | 1 | 1 | 1 | 1 | 1 | 0 | 1 | 14 |
| Dogas et al. [40] | 1 | 0 | 1 | 1 | 1 | 1 | 0 | 1 | 0 | 1 | 0 | 1 | 0 | 1 | 1 | 0 | 10 |
| Dun et al. [41] | 1 | 1 | 1 | 1 | 1 | 1 | 0 | 1 | 1 | 1 | 1 | 1 | 1 | 1 | 1 | 1 | 15 |
| Dunton et al. [42] | 1 | 1 | 1 | 1 | 1 | 1 | 0 | 1 | 0 | 1 | 1 | 1 | 1 | 1 | 1 | 1 | 14 |
| Ellis et al. [43] | 1 | 1 | 1 | 1 | 1 | 1 | 0 | 1 | 0 | 1 | 0 | 1 | 0 | 0 | 1 | 1 | 11 |
| Elran-Barak et al. [44] | 1 | 1 | 1 | 1 | 0 | 0 | 0 | 1 | 0 | 1 | 0 | 0 | 1 | 1 | 1 | 1 | 10 |
| Esain et al. [45] | 1 | 1 | 1 | 1 | 1 | 1 | 1 | 1 | 0 | 1 | 1 | 1 | 1 | 1 | 1 | 1 | 15 |
| Fernandez-Garcia et al. [46] | 1 | 1 | 1 | 1 | 1 | 1 | 0 | 0 | 0 | 1 | 0 | 1 | 1 | 0 | 0 | 1 | 10 |
| Feter et al. [47] | 1 | 1 | 1 | 1 | 1 | 1 | 1 | 1 | 0 | 1 | 0 | 1 | 1 | 1 | 1 | 1 | 14 |
| Flanagan et al. [48] | 1 | 1 | 1 | 1 | 1 | 1 | 0 | 1 | 0 | 1 | 1 | 1 | 1 | 1 | 0 | 0 | 12 |
| Francisco-Pascual et al. [49] | 1 | 1 | 1 | 1 | 1 | 1 | 1 | 1 | 1 | 1 | 1 | 1 | 0 | 1 | 1 | 1 | 15 |
| Franco et al. [50] | 1 | 1 | 1 | 0 | 1 | 1 | 0 | 1 | 0 | 1 | 1 | 1 | 1 | 1 | 0 | 0 | 15 |
| Franco et al. [51] | 1 | 1 | 1 | 1 | 1 | 1 | 0 | 1 | 0 | 1 | 0 | 1 | 1 | 1 | 1 | 1 | 13 |
| Füzeki et al. [52] | 1 | 1 | 1 | 1 | 1 | 1 | 0 | 1 | 0 | 1 | 1 | 1 | 1 | 1 | 1 | 1 | 14 |
| Füzéki et al. [53] | 1 | 1 | 1 | 1 | 1 | 1 | 1 | 1 | 0 | 1 | 1 | 1 | 1 | 1 | 1 | 1 | 15 |
| Gallè et al. [54] | 1 | 1 | 1 | 1 | 1 | 1 | 1 | 1 | 0 | 1 | 1 | 1 | 1 | 1 | 1 | 1 | 15 |
| Genin et al. [55] | 1 | 1 | 1 | 1 | 1 | 1 | 1 | 1 | 0 | 1 | 1 | 1 | 1 | 1 | 1 | 1 | 15 |
| Gierc et al. [56] | 1 | 1 | 1 | 1 | 1 | 1 | 0 | 1 | 0 | 1 | 1 | 1 | 1 | 1 | 1 | 1 | 15 |
| Gilic et al. [57] | 1 | 1 | 1 | 1 | 1 | 1 | 0 | 1 | 0 | 1 | 1 | 1 | 1 | 1 | 1 | 0 | 13 |
| Giustino et al. [58] | 1 | 1 | 1 | 1 | 1 | 1 | 0 | 1 | 0 | 1 | 1 | 1 | 1 | 1 | 1 | 0 | 13 |
| Gjaka et al. [59] | 1 | 1 | 1 | 1 | 1 | 1 | 0 | 1 | 1 | 1 | 1 | 1 | 1 | 1 | 1 | 1 | 15 |
| Haider et al. [60] | 1 | 1 | 1 | 1 | 1 | 1 | 0 | 1 | 0 | 1 | 0 | 0 | 1 | 0 | 0 | 1 | 10 |
| Han et al. [61] | 1 | 1 | 1 | 1 | 1 | 1 | 1 | 1 | 0 | 1 | 1 | 1 | 1 | 1 | 0 | 0 | 13 |
| Hargreaves et al. [62] | 1 | 1 | 1 | 1 | 1 | 1 | 0 | 1 | 0 | 1 | 1 | 1 | 1 | 1 | 1 | 0 | 13 |
| Harrison et al. [63] | 1 | 1 | 1 | 1 | 1 | 1 | 1 | 1 | 0 | 1 | 1 | 1 | 1 | 1 | 1 | 1 | 15 |
| He et al. [64] | 1 | 1 | 0 | 1 | 1 | 1 | 0 | 1 | 0 | 1 | 1 | 1 | 1 | 0 | 0 | 1 | 11 |
| Hemphill et al. [65] | 1 | 1 | 1 | 1 | 1 | 1 | 0 | 1 | 1 | 1 | 1 | 1 | 1 | 1 | 1 | 1 | 15 |
| Henriksen et al. [66] | 1 | 1 | 1 | 1 | 1 | 1 | 1 | 1 | 0 | 1 | 1 | 1 | 1 | 1 | 1 | 1 | 15 |
| Hermassi et al. [67] | 1 | 1 | 1 | 1 | 1 | 1 | 1 | 1 | 1 | 1 | 1 | 1 | 1 | 1 | 1 | 1 | 16 |
| Hermassi et al. [68] | 1 | 1 | 1 | 1 | 1 | 1 | 1 | 1 | 0 | 1 | 1 | 1 | 1 | 1 | 1 | 1 | 15 |
| Huber et al. [69] | 1 | 1 | 0 | 1 | 1 | 1 | 0 | 1 | 0 | 1 | 1 | 1 | 1 | 0 | 1 | 1 | 12 |
| Huber et al. [70] | 0 | 1 | 1 | 0 | 1 | 0 | 0 | 1 | 0 | 0 | 1 | 0 | 1 | 0 | 0 | 0 | 6 |
| Hume et al. [71] | 0 | 1 | 1 | 0 | 1 | 0 | 0 | 0 | 0 | 0 | 1 | 0 | 1 | 0 | 0 | 0 | 5 |
| Imaz-Aramburu et al. [72] | 1 | 1 | 1 | 1 | 1 | 1 | 1 | 1 | 0 | 1 | 1 | 1 | 1 | 0 | 0 | 0 | 12 |
| Jalal et al. [73] | 1 | 1 | 1 | 1 | 1 | 1 | 1 | 1 | 0 | 1 | 1 | 1 | 1 | 1 | 0 | 0 | 13 |
| Janssen et al. [74] | 1 | 1 | 0 | 1 | 1 | 1 | 0 | 1 | 0 | 1 | 0 | 1 | 1 | 0 | 0 | 1 | 10 |
| Jia et al. [75] | 1 | 1 | 1 | 1 | 1 | 1 | 0 | 1 | 0 | 1 | 1 | 1 | 1 | 0 | 1 | 1 | 13 |
| Joseph et al. [76] | 1 | 1 | 1 | 1 | 1 | 1 | 0 | 1 | 0 | 1 | 1 | 1 | 1 | 1 | 1 | 1 | 14 |
| Karuc et al. [77] | 1 | 1 | 1 | 1 | 1 | 1 | 1 | 1 | 0 | 1 | 0 | 1 | 1 | 1 | 0 | 1 | 13 |
| Kontsevaya et al. [78] | 1 | 1 | 1 | 1 | 1 | 1 | 0 | 1 | 1 | 1 | 1 | 1 | 1 | 1 | 1 | 1 | 15 |
| Koohsari et al. [79] | 1 | 1 | 1 | 1 | 1 | 1 | 1 | 1 | 0 | 1 | 1 | 1 | 1 | 1 | 1 | 1 | 15 |
| Kouis et al. [80] | 1 | 1 | 1 | 1 | 1 | 1 | 0 | 1 | 0 | 0 | 1 | 0 | 1 | 0 | 0 | 0 | 9 |
| Kowalsky et al. [81] | 1 | 1 | 1 | 1 | 1 | 1 | 1 | 1 | 0 | 1 | 1 | 1 | 1 | 1 | 0 | 0 | 13 |
| LaCaille et al. [82] | 1 | 1 | 1 | 1 | 1 | 1 | 1 | 1 | 0 | 1 | 1 | 1 | 1 | 1 | 1 | 1 | 15 |
| Lawrence et al. [83] | 1 | 1 | 0 | 1 | 1 | 1 | 0 | 1 | 0 | 1 | 0 | 1 | 1 | 0 | 0 | 1 | 10 |
| Leppä et al. [84] | 1 | 1 | 1 | 1 | 1 | 1 | 1 | 1 | 0 | 1 | 1 | 1 | 1 | 1 | 1 | 1 | 15 |
| Li et al. [85] | 1 | 1 | 1 | 1 | 1 | 1 | 1 | 1 | 0 | 1 | 1 | 1 | 1 | 1 | 1 | 1 | 15 |
| Lopez-Bueno et al. [86] | 1 | 1 | 1 | 1 | 1 | 1 | 0 | 1 | 0 | 1 | 1 | 1 | 1 | 1 | 1 | 1 | 14 |
| Lopez-Bueno et al. [87] | 1 | 1 | 1 | 1 | 1 | 1 | 0 | 1 | 0 | 1 | 0 | 1 | 1 | 0 | 1 | 1 | 12 |
| Lopez-Bueno et al. [88] | 1 | 1 | 1 | 1 | 1 | 1 | 0 | 1 | 0 | 1 | 0 | 1 | 1 | 0 | 1 | 1 | 12 |
| Lopez-Gil et al. [89] | 1 | 1 | 1 | 1 | 1 | 1 | 0 | 1 | 0 | 1 | 0 | 1 | 1 | 0 | 1 | 1 | 12 |
| Lopez-Sanchez et al. [90] | 1 | 1 | 1 | 1 | 1 | 1 | 0 | 1 | 0 | 1 | 0 | 1 | 1 | 0 | 1 | 1 | 12 |
| Maher et al. [91] | 1 | 1 | 1 | 1 | 1 | 1 | 0 | 1 | 0 | 1 | 0 | 1 | 1 | 1 | 1 | 1 | 13 |
| Makizako et al. [92] | 1 | 1 | 1 | 1 | 0 | 1 | 0 | 1 | 0 | 1 | 0 | 1 | 1 | 0 | 1 | 1 | 11 |
| Malanchini et al. [93] | 1 | 1 | 1 | 1 | 1 | 1 | 0 | 1 | 0 | 1 | 1 | 1 | 1 | 0 | 0 | 1 | 12 |
| Maltagliati et al. [94] | 1 | 1 | 1 | 0 | 1 | 1 | 1 | 1 | 0 | 1 | 1 | 1 | 1 | 1 | 0 | 1 | 13 |
| Maltoni et al. [95] | 1 | 1 | 1 | 1 | 1 | 1 | 0 | 1 | 1 | 1 | 1 | 1 | 1 | 1 | 1 | 0 | 14 |
| Marco-Ahulla et al. [96] | 1 | 1 | 0 | 1 | 1 | 0 | 1 | 1 | 0 | 1 | 1 | 1 | 1 | 1 | 0 | 1 | 12 |
| Martinez-de-Quel et al. [97] | 1 | 1 | 0 | 1 | 1 | 1 | 1 | 1 | 0 | 1 | 0 | 1 | 1 | 0 | 0 | 1 | 11 |
| Mascherini et al. [98] | 1 | 1 | 1 | 1 | 1 | 1 | 0 | 1 | 0 | 1 | 1 | 1 | 1 | 1 | 0 | 0 | 12 |
| Mason et al. [99] | 1 | 1 | 1 | 0 | 1 | 1 | 0 | 1 | 0 | 1 | 1 | 1 | 1 | 1 | 1 | 0 | 12 |
| Maugeri et al. [100] | 1 | 1 | 0 | 1 | 1 | 1 | 0 | 1 | 0 | 1 | 0 | 1 | 1 | 1 | 1 | 1 | 12 |
| Mazo et al. [101] | 1 | 1 | 1 | 1 | 1 | 1 | 1 | 1 | 0 | 1 | 1 | 1 | 1 | 1 | 1 | 1 | 15 |
| McCarthy et al. [102] | 1 | 1 | 0 | 1 | 1 | 1 | 0 | 1 | 0 | 1 | 1 | 1 | 1 | 1 | 1 | 1 | 13 |
| Medrano et al. [103] | 1 | 1 | 1 | 1 | 1 | 1 | 1 | 1 | 1 | 1 | 0 | 1 | 1 | 1 | 1 | 1 | 15 |
| Meyer et al. [104] | 1 | 1 | 1 | 1 | 1 | 1 | 0 | 1 | 0 | 1 | 0 | 1 | 1 | 1 | 1 | 1 | 13 |
| Mirhajianmoghadam et al. [105] | 1 | 1 | 1 | 1 | 1 | 1 | 1 | 1 | 1 | 1 | 1 | 1 | 1 | 1 | 1 | 1 | 16 |
| Mishra et al. [106] | 1 | 1 | 1 | 1 | 1 | 1 | 0 | 1 | 1 | 1 | 1 | 1 | 1 | 1 | 0 | 1 | 14 |
| Miyahara et al. [107] | 1 | 1 | 1 | 1 | 1 | 1 | 1 | 1 | 1 | 1 | 1 | 1 | 1 | 0 | 0 | 0 | 13 |
| Mon-Lopez et al. [108] | 1 | 1 | 0 | 1 | 1 | 1 | 0 | 1 | 0 | 1 | 1 | 1 | 1 | 1 | 1 | 1 | 13 |
| Moura et al. [109] | 1 | 1 | 1 | 1 | 1 | 1 | 1 | 1 | 0 | 1 | 1 | 1 | 1 | 1 | 1 | 1 | 15 |
| Mutz-Reimers et al. [110] | 1 | 1 | 1 | 1 | 1 | 1 | 1 | 1 | 1 | 1 | 1 | 1 | 1 | 1 | 1 | 1 | 16 |
| Nascimento et al. [111] | 1 | 1 | 1 | 1 | 1 | 1 | 1 | 1 | 0 | 1 | 1 | 1 | 1 | 1 | 0 | 1 | 14 |
| Natalucci et al. [112] | 1 | 1 | 1 | 1 | 1 | 1 | 1 | 1 | 1 | 1 | 1 | 1 | 1 | 1 | 1 | 1 | 16 |
| Nathan et al. [113] | 1 | 1 | 1 | 1 | 1 | 1 | 0 | 1 | 0 | 1 | 1 | 1 | 0 | 1 | 1 | 1 | 13 |
| Naughton et al. [114] | 1 | 1 | 1 | 1 | 1 | 1 | 1 | 1 | 0 | 1 | 1 | 1 | 1 | 1 | 1 | 1 | 15 |
| Okely et al. [115] | 1 | 1 | 1 | 0 | 1 | 1 | 1 | 1 | 0 | 1 | 1 | 1 | 1 | 1 | 0 | 1 | 13 |
| Oliveira et al. [116] | 1 | 1 | 1 | 1 | 1 | 1 | 0 | 0 | 1 | 1 | 1 | 1 | 1 | 1 | 1 | 1 | 14 |
| Ong et al. [117] | 0 | 1 | 0 | 1 | 1 | 1 | 1 | 1 | 0 | 1 | 1 | 1 | 1 | 0 | 0 | 1 | 11 |
| Park et al. [118] | 1 | 1 | 1 | 1 | 1 | 1 | 1 | 0 | 0 | 1 | 1 | 1 | 1 | 1 | 1 | 0 | 13 |
| Perez et al. [119] | 1 | 1 | 1 | 1 | 1 | 1 | 1 | 1 | 0 | 1 | 0 | 1 | 1 | 0 | 1 | 1 | 13 |
| Pisot et al. [120] | 1 | 1 | 1 | 1 | 1 | 1 | 0 | 1 | 0 | 1 | 0 | 1 | 1 | 0 | 1 | 1 | 12 |
| Pla et al. [121] | 1 | 1 | 1 | 1 | 1 | 1 | 1 | 1 | 0 | 1 | 1 | 1 | 1 | 1 | 1 | 1 | 15 |
| Predieri et al. [122] | 1 | 1 | 1 | 1 | 1 | 1 | 1 | 1 | 1 | 1 | 1 | 1 | 1 | 1 | 1 | 1 | 16 |
| Rastogi et al. [123] | 1 | 1 | 1 | 1 | 1 | 1 | 0 | 1 | 0 | 1 | 0 | 1 | 1 | 1 | 1 | 1 | 13 |
| Rhodes et al. [124] | 1 | 1 | 1 | 1 | 1 | 1 | 1 | 1 | 1 | 1 | 1 | 1 | 1 | 1 | 1 | 1 | 16 |
| Ribeiro de Lima et al. [125] | 1 | 1 | 1 | 1 | 1 | 1 | 0 | 1 | 0 | 0 | 1 | 1 | 1 | 1 | 0 | 1 | 12 |
| Richardson et al. [126] | 1 | 1 | 1 | 1 | 1 | 1 | 0 | 1 | 0 | 1 | 0 | 1 | 1 | 0 | 0 | 1 | 11 |
| Roberts-Lewis et al. [127] | 1 | 1 | 0 | 0 | 0 | 1 | 0 | 0 | 0 | 0 | 1 | 0 | 1 | 0 | 0 | 0 | 5 |
| Rodriguez-Larrad et al. [128] | 1 | 1 | 1 | 1 | 1 | 1 | 0 | 1 | 0 | 1 | 0 | 1 | 1 | 0 | 1 | 1 | 12 |
| Romero-Blanco et al. [129] | 1 | 1 | 1 | 1 | 1 | 1 | 0 | 1 | 0 | 1 | 1 | 1 | 1 | 0 | 1 | 1 | 13 |
| Rowlands et al. [130] | 1 | 1 | 1 | 1 | 1 | 1 | 0 | 1 | 0 | 1 | 1 | 1 | 1 | 1 | 1 | 0 | 13 |
| Ruiz-Roso et al. [131] | 1 | 1 | 1 | 1 | 1 | 1 | 0 | 1 | 0 | 1 | 1 | 1 | 1 | 0 | 1 | 1 | 13 |
| Sadarangani et al. [132] | 1 | 1 | 1 | 1 | 1 | 1 | 1 | 1 | 0 | 1 | 1 | 1 | 1 | 1 | 1 | 1 | 15 |
| Salman et al. [133] | 1 | 1 | 1 | 1 | 1 | 1 | 1 | 1 | 0 | 1 | 1 | 1 | 1 | 1 | 1 | 1 | 15 |
| Sanudo et al. [134] | 1 | 1 | 1 | 1 | 1 | 1 | 0 | 1 | 0 | 1 | 0 | 1 | 1 | 0 | 0 | 1 | 11 |
| Sasaki et al. [135] | 1 | 1 | 1 | 1 | 1 | 1 | 0 | 1 | 1 | 1 | 1 | 1 | 1 | 0 | 1 | 1 | 14 |
| Sassone et al. [136] | 1 | 1 | 0 | 0 | 1 | 1 | 0 | 1 | 0 | 1 | 1 | 0 | 1 | 1 | 0 | 0 | 9 |
| Savage et al. [137] | 1 | 1 | 1 | 1 | 0 | 1 | 0 | 1 | 0 | 1 | 1 | 1 | 1 | 0 | 1 | 1 | 12 |
| Schlichtiger et al. [138] | 1 | 1 | 1 | 1 | 1 | 1 | 0 | 1 | 0 | 1 | 0 | 1 | 1 | 0 | 0 | 1 | 11 |
| Schmidt & Pawlowski [139] | 1 | 1 | 1 | 1 | 1 | 1 | 1 | 1 | 0 | 1 | 1 | 1 | 1 | 1 | 1 | 1 | 15 |
| Schmidt et al. [140] | 0 | 1 | 0 | 0 | 1 | 1 | 0 | 1 | 1 | 1 | 0 | 1 | 1 | 1 | 0 | 1 | 10 |
| Sekulic et al. [141] | 1 | 1 | 1 | 1 | 1 | 1 | 0 | 1 | 0 | 1 | 0 | 1 | 1 | 0 | 1 | 1 | 12 |
| Shin et al. [142] | 1 | 1 | 1 | 1 | 1 | 1 | 1 | 1 | 0 | 1 | 1 | 1 | 1 | 1 | 1 | 0 | 14 |
| Sidebottom et al. [143] | 1 | 1 | 1 | 1 | 1 | 1 | 1 | 1 | 0 | 1 | 1 | 1 | 1 | 1 | 0 | 0 | 13 |
| Silva Santos et al. [144] | 1 | 1 | 1 | 1 | 1 | 1 | 1 | 1 | 0 | 1 | 1 | 1 | 1 | 0 | 0 | 1 | 13 |
| Silva-Batista et al. [145] | 1 | 1 | 1 | 1 | 1 | 1 | 1 | 1 | 0 | 1 | 1 | 1 | 1 | 1 | 1 | 0 | 14 |
| Song et al. [146] | 1 | 1 | 1 | 1 | 1 | 1 | 1 | 1 | 1 | 1 | 0 | 1 | 1 | 1 | 0 | 1 | 14 |
| Souza et al. [147] | 1 | 1 | 1 | 1 | 1 | 1 | 0 | 1 | 0 | 1 | 1 | 1 | 1 | 1 | 0 | 0 | 12 |
| Srivastav et al. [148] | 1 | 1 | 0 | 1 | 1 | 1 | 0 | 1 | 0 | 1 | 0 | 1 | 1 | 1 | 0 | 1 | 11 |
| Strutt et al. [149] | 1 | 1 | 1 | 1 | 1 | 1 | 1 | 1 | 0 | 1 | 1 | 0 | 1 | 1 | 0 | 0 | 12 |
| Szabo et al. [150] | 0 | 1 | 1 | 1 | 1 | 1 | 0 | 1 | 0 | 1 | 0 | 1 | 1 | 0 | 0 | 1 | 10 |
| Theis et al. [151] | 1 | 1 | 1 | 1 | 1 | 1 | 0 | 1 | 0 | 1 | 0 | 1 | 1 | 1 | 1 | 1 | 13 |
| Tornaghi et al. [152] | 1 | 1 | 0 | 0 | 1 | 0 | 0 | 0 | 0 | 1 | 0 | 1 | 1 | 0 | 0 | 1 | 7 |
| Ugbolue et al. [153] | 1 | 1 | 0 | 0 | 1 | 1 | 0 | 1 | 0 | 1 | 0 | 1 | 1 | 0 | 0 | 1 | 9 |
| van Bakel et al. [154] | 1 | 1 | 1 | 1 | 1 | 1 | 1 | 1 | 1 | 1 | 1 | 1 | 1 | 1 | 0 | 1 | 15 |
| Vetrovsky et al. [155] | 1 | 1 | 1 | 1 | 1 | 1 | 1 | 1 | 0 | 1 | 1 | 1 | 1 | 0 | 1 | 1 | 14 |
| Wang et al. [156] | 1 | 1 | 1 | 1 | 1 | 1 | 1 | 1 | 0 | 1 | 1 | 1 | 1 | 1 | 0 | 1 | 14 |
| Wang et al. [157] | 1 | 1 | 1 | 1 | 1 | 1 | 1 | 1 | 1 | 1 | 1 | 1 | 1 | 1 | 0 | 0 | 14 |
| Wang et al. [158] | 1 | 1 | 1 | 1 | 1 | 1 | 1 | 1 | 1 | 1 | 1 | 1 | 1 | 1 | 1 | 1 | 16 |
| Wang J et al. [159] | 1 | 1 | 1 | 1 | 1 | 1 | 0 | 1 | 0 | 1 | 1 | 1 | 1 | 1 | 1 | 0 | 13 |
| Werneck et al. [160] | 0 | 1 | 0 | 1 | 0 | 1 | 0 | 0 | 1 | 1 | 1 | 1 | 1 | 1 | 1 | 1 | 11 |
| Wilke et al. [161] | 1 | 1 | 1 | 0 | 1 | 1 | 0 | 1 | 0 | 1 | 1 | 1 | 1 | 1 | 0 | 0 | 11 |
| Woodruff et al. [162] | 1 | 1 | 0 | 1 | 1 | 0 | 0 | 1 | 0 | 1 | 0 | 1 | 1 | 0 | 0 | 1 | 9 |
| Wunsch et al. [163] | 1 | 0 | 1 | 1 | 1 | 1 | 0 | 1 | 1 | 1 | 0 | 1 | 1 | 1 | 0 | 1 | 12 |
| Yamada et al. [164] | 1 | 1 | 1 | 1 | 1 | 1 | 0 | 1 | 0 | 1 | 0 | 1 | 1 | 0 | 0 | 1 | 11 |
| Yamada M et al. [165] | 1 | 1 | 1 | 1 | 1 | 1 | 0 | 1 | 0 | 1 | 1 | 1 | 1 | 1 | 1 | 1 | 14 |
| Yang et al. [166] | 1 | 1 | 1 | 1 | 1 | 1 | 0 | 0 | 0 | 1 | 1 | 1 | 1 | 0 | 0 | 1 | 11 |
| Yang et al. [167] | 1 | 1 | 1 | 1 | 1 | 1 | 0 | 1 | 0 | 1 | 0 | 1 | 1 | 0 | 1 | 1 | 12 |
| Yang et al. [168] | 1 | 1 | 1 | 1 | 1 | 1 | 0 | 1 | 0 | 1 | 1 | 1 | 1 | 1 | 1 | 1 | 14 |
| Zenic et al. [169] | 1 | 1 | 0 | 0 | 0 | 0 | 0 | 1 | 0 | 1 | 0 | 1 | 1 | 0 | 1 | 1 | 8 |
| Zheng et al. [170] | 1 | 1 | 1 | 1 | 1 | 1 | 0 | 1 | 0 | 1 | 0 | 1 | 1 | 0 | 0 | 1 | 11 |
| Zhu et al. [171] | 1 | 1 | 1 | 1 | 1 | 1 | 0 | 0 | 0 | 1 | 0 | 1 | 0 | 0 | 0 | 1 | 9 |
| Zinner et al. [172] | 1 | 1 | 1 | 1 | 1 | 1 | 0 | 1 | 0 | 1 | 1 | 1 | 1 | 0 | 0 | 1 | 12 |
